# Effects of canagliflozin on human myocardial redox signalling: clinical implications

**DOI:** 10.1093/eurheartj/ehab420

**Published:** 2021-07-19

**Authors:** Hidekazu Kondo, Ioannis Akoumianakis, Ileana Badi, Nadia Akawi, Christos P Kotanidis, Murray Polkinghorne, Ilaria Stadiotti, Elena Sommariva, Alexios S Antonopoulos, Maria C Carena, Evangelos K Oikonomou, Elsa Mauricio Reus, Rana Sayeed, George Krasopoulos, Vivek Srivastava, Shakil Farid, Surawee Chuaiphichai, Cheerag Shirodaria, Keith M Channon, Barbara Casadei, Charalambos Antoniades

**Affiliations:** Division of Cardiovascular Medicine, Radcliffe Department of Medicine, University of Oxford, L6 West Wing, John Radcliffe Hospital, Headley Way, Oxford OX3 9DU, UK; Department of Cardiology and Clinical Examination, Faculty of Medicine, Oita University, 1-1 Idaigaoka, Hasama, Yufu, Oita 879-5593, Japan; Division of Cardiovascular Medicine, Radcliffe Department of Medicine, University of Oxford, L6 West Wing, John Radcliffe Hospital, Headley Way, Oxford OX3 9DU, UK; Division of Cardiovascular Medicine, Radcliffe Department of Medicine, University of Oxford, L6 West Wing, John Radcliffe Hospital, Headley Way, Oxford OX3 9DU, UK; Division of Cardiovascular Medicine, Radcliffe Department of Medicine, University of Oxford, L6 West Wing, John Radcliffe Hospital, Headley Way, Oxford OX3 9DU, UK; Department of Genetics and Genomics, College of Medicine and Health Sciences, United Arab Emirates University, Khalifa Ibn Zayed Street, Al Maqam, Al-Ain, P.O. Box 17666, United Arab Emirates; Division of Cardiovascular Medicine, Radcliffe Department of Medicine, University of Oxford, L6 West Wing, John Radcliffe Hospital, Headley Way, Oxford OX3 9DU, UK; Division of Cardiovascular Medicine, Radcliffe Department of Medicine, University of Oxford, L6 West Wing, John Radcliffe Hospital, Headley Way, Oxford OX3 9DU, UK; Unit of Vascular Biology and Regenerative Medicine, Centro Cardiologico Monzino IRCCS, via Carlo Parea 4, 20138, Milan, Italy; Unit of Vascular Biology and Regenerative Medicine, Centro Cardiologico Monzino IRCCS, via Carlo Parea 4, 20138, Milan, Italy; Division of Cardiovascular Medicine, Radcliffe Department of Medicine, University of Oxford, L6 West Wing, John Radcliffe Hospital, Headley Way, Oxford OX3 9DU, UK; Division of Cardiovascular Medicine, Radcliffe Department of Medicine, University of Oxford, L6 West Wing, John Radcliffe Hospital, Headley Way, Oxford OX3 9DU, UK; Division of Cardiovascular Medicine, Radcliffe Department of Medicine, University of Oxford, L6 West Wing, John Radcliffe Hospital, Headley Way, Oxford OX3 9DU, UK; Division of Cardiovascular Medicine, Radcliffe Department of Medicine, University of Oxford, L6 West Wing, John Radcliffe Hospital, Headley Way, Oxford OX3 9DU, UK; Oxford University Hospitals NHS Trust, Headley Way, Oxford OX3 9DU, UK; Oxford University Hospitals NHS Trust, Headley Way, Oxford OX3 9DU, UK; Oxford University Hospitals NHS Trust, Headley Way, Oxford OX3 9DU, UK; Oxford University Hospitals NHS Trust, Headley Way, Oxford OX3 9DU, UK; Division of Cardiovascular Medicine, Radcliffe Department of Medicine, University of Oxford, L6 West Wing, John Radcliffe Hospital, Headley Way, Oxford OX3 9DU, UK; Caristo Diagnostics, 1st Floor, New Barclay House, 234 Botley Rd, Oxford OX2 0HP, UK; Division of Cardiovascular Medicine, Radcliffe Department of Medicine, University of Oxford, L6 West Wing, John Radcliffe Hospital, Headley Way, Oxford OX3 9DU, UK; Oxford University Hospitals NHS Trust, Headley Way, Oxford OX3 9DU, UK; Division of Cardiovascular Medicine, Radcliffe Department of Medicine, University of Oxford, L6 West Wing, John Radcliffe Hospital, Headley Way, Oxford OX3 9DU, UK; Division of Cardiovascular Medicine, Radcliffe Department of Medicine, University of Oxford, L6 West Wing, John Radcliffe Hospital, Headley Way, Oxford OX3 9DU, UK; Oxford University Hospitals NHS Trust, Headley Way, Oxford OX3 9DU, UK; Acute Vascular Imaging Centre, University of Oxford, Headley Way, Oxford OX3 9DU, UK

**Keywords:** SGLT2 inhibitor, SGLT1, Myocardial redox state, NADPH oxidase activity, NOS coupling, AMPK

## Abstract

**Aims:**

Recent clinical trials indicate that sodium-glucose cotransporter 2 (SGLT2) inhibitors improve cardiovascular outcomes in heart failure patients, but the underlying mechanisms remain unknown. We explored the direct effects of canagliflozin, an SGLT2 inhibitor with mild SGLT1 inhibitory effects, on myocardial redox signalling in humans.

**Methods and results:**

Study 1 included 364 patients undergoing cardiac surgery. Right atrial appendage biopsies were harvested to quantify superoxide ($O_{2}^{.-}$) sources and the expression of inflammation, fibrosis, and myocardial stretch genes. In Study 2, atrial tissue from 51 patients was used *ex vivo* to study the direct effects of canagliflozin on NADPH oxidase activity and nitric oxide synthase (NOS) uncoupling. Differentiated H9C2 and primary human cardiomyocytes (hCM) were used to further characterize the underlying mechanisms (Study 3). *SGLT1* was abundantly expressed in human atrial tissue and hCM, contrary to *SGLT2*. Myocardial *SGLT1* expression was positively associated with $O_{2}^{.-}$ production and pro-fibrotic, pro-inflammatory, and wall stretch gene expression. Canagliflozin reduced NADPH oxidase activity via AMP kinase (AMPK)/Rac1signalling and improved NOS coupling via increased tetrahydrobiopterin bioavailability *ex vivo* and *in vitro*. These were attenuated by knocking down *SGLT1* in hCM. Canagliflozin had striking *ex vivo* transcriptomic effects on myocardial redox signalling, suppressing apoptotic and inflammatory pathways in hCM.

**Conclusions:**

We demonstrate for the first time that canagliflozin suppresses myocardial NADPH oxidase activity and improves NOS coupling via SGLT1/AMPK/Rac1 signalling, leading to global anti-inflammatory and anti-apoptotic effects in the human myocardium. These findings reveal a novel mechanism contributing to the beneficial cardiac effects of canagliflozin.

## Graphical Abstract

**Figure:**
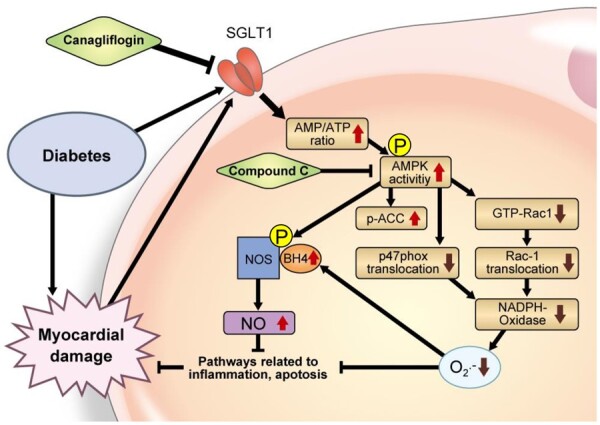
Proposed mechanism of canagliflozin-induced improvement of myocardial redox state. Canagliflozin increases intracellular AMP/ATP ratio through inhibition of SGLT1, which can activate AMPK/NOS signalling and increase NO that suppresses pro-inflammatory signalling. AMPK activation also inhibits activation of Rac1 and membrane translocation of Rac1 and p47^phox^, which decrease NADPH oxidase activity and superoxide ($O_{2}^{.-}$) production, attenuates inflammatory and apoptotic pathways and increasing the bioavailability of tetrahydrobiopterin (BH4), a key factor for NOS coupling.

##  

**See page 4961 for the editorial comment for this article ‘Canagliflozin and myocardial oxidative stress: SGLT1 inhibition takes centre stage’, by G.S. Schiattarella and D. Bode, https://doi.org/10.1093/eurheartj/ehab519.**

**Translational perspective**

Sodium-glucose cotransporter 2 (SGLT2) inhibitors are a novel calls of anti-diabetic drugs that have recently emerged as cardioprotective agents in heart failure, even in the absence of diabetes. Despite their clinical benefit, the underlying effects on the human myocardium remain largely unexplored. In this work, we demonstrate a novel, SGLT1-mediated effect of canagliflozin, a clinically used SGLT2 inhibitor, on myocardial biology affecting its redox state, inflammation, fibrosis, and apoptosis in humans. Our work suggests novel cellular mechanisms underlying the observed clinical benefits of SGLT2 inhibitors; further characterization of the mechanistic effects of the SGLT2 inhibitor drug class on the human myocardium will broaden their use and indications in cardiovascular disease more efficiently.

## Introduction

Sodium-glucose cotransporter 2 (SGLT2) inhibitors comprise an anti-hyperglycaemic drug class regulating plasma glucose by inhibiting glucose reabsorption through SGLT2 blockade in the proximal renal tubules.^1^ Clinically available SGLT2 inhibitors also display variable affinity to SGLT1.^2^ While SGLT2 is the main responsible for glucose reabsorption from the glomerular filtrate, SGLT1, which is essential for the fast uptake of glucose and galactose in the intestine, plays a minor role in renal reabsorption. These two sodium-glucose cotransporters have different affinity and capacity for glucose transport and diverse expression patterns in the renal proximal tubule as well as in other organs.^3^ The cardioprotective effects of SGLT2 inhibitors have been demonstrated in recent clinical trials (EMPA-REG OUTCOME, CANVAS, DECLARE-TIMI 58) in diabetic^4–7^ as well as non-diabetic patients with heart failure (DAPA-HF).^8^^,^^9^ Accordingly, the 2019 ESC guidelines on diabetes, pre-diabetes, and cardiovascular diseases recommend empagliflozin, canagliflozin, or dapagliflozin in patients with type 2 diabetes mellitus and cardiovascular disease, or at high/very high cardiovascular risk, to reduce cardiovascular events, as well as in diabetic patients to lower the risk of heart failure hospitalization.^10^ However, the mechanisms behind these effects remain unclear.

Limited evidence suggests that SGLT2 inhibitors have direct cardioprotective effects that extend beyond their systemic glucose-lowering function. Empagliflozin attenuates cardiac fibrosis in diabetic mice^11^ and preserves myocardial function in a mouse model of pressure overload-induced heart failure.^12^ Canagliflozin activates AMP kinase (AMPK), whose cardioprotective effects are well established, by inhibiting mitochondrial function and increasing cellular AMP levels *in vitro.* ^13^ However, several aspects of the direct effects of SGLT2 inhibition on the human heart remain unclear.^14^

Sustained oxidative stress is central to the pathogenesis of cardiac diseases, such as heart failure^15^ and atrial fibrillation.^16^^,^^17^ NADPH oxidases (Nox) are major sources of superoxide anions ($O_{2}^{.-}$) in the human heart, and the activity of Nox1 and Nox2 isoforms is dependent on activation and membrane translocation of the GTPase Rac1 and the presence of p47^phox.18^ On the other hand, nitric oxide synthases (NOSs) are redox-related enzymes responsible for nitric oxide (NO) synthesis in the human heart.^19^ In disease states such as long-standing persistent atrial fibrillation, myocardial NOS appears to be uncoupled, therefore producing $O_{2}^{.-}$ rather than NO as a result of oxidative depletion of its co-factor tetrahydrobiopterin (BH4).^20^ Similar NOS uncoupling is also observed in various diseases like diabetes and hypertension.^21^ However, despite the importance of myocardial redox state regulation in cardiac biology, the putative antioxidant effects on the human heart of different SGLT2 inhibitors have not been well investigated so far.^14^

In this study, we first explored the expression profiles of SGLT2 and SGLT1 in the human heart to determine the molecular target of direct SGLT1/2 inhibition in the human atrial myocardium. Secondly, we evaluated the direct effects of the SGLT2-specific inhibitor empagliflozin and the dual SGLT1/2 inhibitor canagliflozin on myocardial redox signalling in the human heart.

## Methods

### Study population

The study population consisted of patients undergoing cardiac surgery, all of whom were under the Oxford Heart Vessels and Fat (ox-HVF) programme (www.oxhvf.com) at Oxford University Hospitals NHS Foundation Trust, UK. The patient’s demographics, indication for surgery, and medication are presented in *Table 1*.

**Table 1:** Demographic characteristics of the study participants

	Clinical study (Study 1)	Ex-vivo study (Study 2)
Patients, *n*	364	51
Age (years)	69 [60–75]	66 [57.25–74.00]
Male sex	301 (82.70)	41 (80.40)
Hypertension	265 (72.80)	36 (70.60)
Hyperlipidaemia	282 (77.50)	34 (66.70)
T2DM	69 (19.00)	9 (17.65)
Smoking		
Active	193 (53)	24 (47.10)
Past	30 (8.2)	3 (5.90)
CrCl (mL/min/1.73 m^2^)	88.14 [25.11–108.99]	97.93 [71.96–116.70]
BMI (kg/m^2^)	27.59 [25.10–30.33]	29.26 [26.03–32.44]
CABG	279 (76.65)	43 (84.31)
AVR due to AR/AS	58 (15.93)	6 (11.77)
MVR due to MR/MS	27 (7.42)	2 (3.92)
NYHA class		
I	133 (36.54)	17 (33.34)
II	154 (42.31)	18 (35.29)
III	64 (17.58)	14 (27.45)
IV	13 (3.57)	2 (3.92)
Medication		
Antiplatelet	280 (76.90)	36 (70.59)
ACEi/ARBs	210 (57.70)	28 (54.90)
Statins	298 (81.90)	34 (66.70)
β-blocker	235 (64.60)	21 (41.20)
CCB	85 (23.40)	12 (23.50)
Insulin	24 (6.60)	6 (11.80)
Oral anti-diabetics	47 (12.90)	4 (7.80)

Values are presented as *n* (%) or median [25th–75th percentile].

ACEi, angiotensin-converting enzyme inhibitor; AR, aortic regurgitation; ARB, angiotensin receptor blocker; AS, aortic stenosis; AVR, aortic valve replacement; BMI, body mass index; CABG, coronary artery bypass grafting; CCB, calcium channel blocker; CrCl, creatinine clearance; MR, mitral regurgitation; MS, mitral stenosis; MVR, mitral valve replacement; T2DM, type 2 diabetes mellitus.

Study 1 was used for cohort-wide associations and consisted of 364 patients. Myocardial biopsies were obtained intraoperatively from the right atrial appendage cannulation site, transferred in ice-cold buffer, and processed for $O_{2}^{.-}$ quantification or gene expression studies as described.^22^ The exclusion criteria were any active inflammatory disease (e.g. autoimmune disease in active/relapse phase) or active infectious disease, advanced liver failure (known or suspected cirrhosis) or end-stage renal disease (estimated glomerular filtration rate <15 mL/min), active malignancy (untreated or under treatment) and systemic use of nonsteroidal anti-inflammatory drugs (i.e. cyclooxygenase-2 inhibitors) or antioxidant vitamins. As chronic obstructive pulmonary disease (COPD) has not been previously associated with changes in myocardial oxidative stress, COPD patients were not excluded if they were not receiving systemic anti-inflammatory treatments.

Study 2 included patient samples used for mechanistic *ex vivo* experiments and consisted of 51 prospectively recruited patients undergoing cardiac surgery with the same exclusion criteria as above. Atrial myocardium specimens were collected during surgery, transferred to the lab, and used for *ex vivo* experiments as described.^22^

Hyperlipidaemia and hypertension were defined according to the current European Society Cardiology guidelines.^23^^,^^24^ Diabetes mellitus was defined according to the American Diabetes Association guidelines.^25^ Study protocols were in agreement with the Declaration of Helsinki and all participants had provided written informed consent. The collection of human ventricle biopsy was approved by the Istituto Europeo di Oncologia and Centro Cardiologico IRCCS—Ethics Committee (R1020/19-94 CCM1072). Participant demographic characteristics are reported in *Table 1*.

### Cell culture models

The effects of SGLT1/2 on cardiomyocytes were first screened in the rat cardiac myocyte-derived cell line, H9c2, and the key findings were replicated in human cardiomyocytes (hCM, PromoCell, Heidelberg, Germany). H9c2 cells were differentiated to cardiac myocytes in Dulbecco’s Modified Eagle Medium (glucose 5.5 mM, Sigma-Aldrich, cat. number D6046) supplemented with 1% horse serum (Sigma-Aldrich). Primary hCM isolated from adult heart ventricles were purchased and differentiated to cardiac myocytes in dedicated Myocyte Growth Medium (glucose 5.5 mM) with SupplementMix (PromoCell) for 60 days after reaching 100% confluence. Troponin I expression and myotube formation were used to confirm efficient cardiomyocyte differentiation (Supplementary material online, *Figure S1*).

Both hCM and H9c2 cells were cultured in a high-glucose medium (25 mM) for 72 h before *in vitro* incubations to mimic the human *ex vivo* experimental environment and the *in vivo* diabetes environment, as 25 mM is the most prevalent concentration of glucose used in the literature to mimic diabetic hyperglycaemia in H9c2 cells.^26^ In selected experiments, glucose was replaced by mannitol 25 mM to study the role of the canagliflozin-induced glucose transfer. Cells were treated with canagliflozin 10 μmol/L (Cayman) ± Compound C (CC, an AMPK inhibitor, 10 μmol/L) or carrier (DMSO) for 60 min or 24 h and used for chemiluminescence experiments, gene expression studies and downstream signalling experiments. The specificity of CC as an AMPK inhibitor was confirmed by tracking downstream phosphorylation of its validated surrogate target, acetyl-CoA carboxylase (ACC).

### Human tissue harvesting

Human myocardial biopsies were processed as described (see Supplementary material online, *Data supplement*) .^22^

### Superoxide quantification

$O_{2}^{.-}$ production was measured in human atrial myocardium homogenates and cell lysates using lucigenin (5 μmol/L)-enhanced chemiluminescence evaluating NADPH oxidase activity and NOS coupling status as described (see Supplementary material online, *Data supplement*).^17^^,^^22^^,^^27^

### RNA isolation and quantitative real time-polymerase chain reaction

RNA was purified in a semi-automated way and processed for cDNA generation and gene expression studies as described (see Supplementary material online, *Data supplement*).^28^

### Western blotting

Western immunoblotting was performed on myocardium homogenates and cell lysates (see Supplementary material online, *Data supplement*).

### Rac1 activation assay

Rac1 activation was detected in myocardial homogenates and cell lysates using a commercial kit (Cell Signalling, see Supplementary material online, *Data supplement*).

### Evaluation of myocardial Rac1 and p47^phox^ membrane translocation

Membrane translocation of Rac1 and p47^phox^ in human myocardial tissue was estimated by differential centrifugation of myocardial homogenates to isolate membrane proteins as described (see Supplementary material online, *Data Supplement*).^22^^,^^29^

### Biopterin measurements

BH4, dihydrobiopterin (BH2), and biopterin levels were determined using high-performance liquid chromatography as described (see Supplementary material online, *Data Supplement*).^27^

### Oxidative fluorescent microtopography

*In situ* $O_{2}^{.-}$ production was determined in human atrial myocardium cryosections and hCMs with an oxidative fluorescent dye (see Supplementary material online, *Data Supplement*).^22^

### TUNEL assay

Apoptotic cells in human myocardial sample sections were detected with the *in situ* terminal deoxynucleotidyl transferase-mediated dUTP nick end-labelling (TUNEL) method using a commercially available kit (see Supplementary material online, *Data Supplement*).

### JC-10 mitochondrial membrane potential assay

The mitochondrial function of samples derived from human atrial myocardium was analysed by means of a commercially available kit (see Supplementary material online, *Data Supplement*).

### Intracellular ADP and ATP measurement

Adenosine triphosphate/Adenosine diphosphate (ATP/ADP) ratio was quantified in hCM by a commercial kit (see Supplementary material online, *Data Supplement*).

### *SGLT1* siRNA transfection studies

*SGLT1* siRNA and negative control siRNA (Thermo Fisher Scientific) were used to knock down SGLT1 in hCMs and in differentiated H9c2 cells by lipofectamine RNAiMax and BlockiT Alexa Fluor Red Fluorescent control as a positive transfection control (see Supplementary material online, *Data Supplement*).

### Transcriptome profiling of canagliflozin-treated human cardiomyocytes

#### Treatments and RNA extraction

Human cardiomyocytes were incubated 24 h with 10 μmol/L canagliflozin or DMSO. RNA was extracted with the RNeasy Micro/Mini kit (Qiagen), and samples were purified with an RNA purification kit (ReliaPrep RNA Clean-up and Concentration System, Promega), achieving 260/280 ratio > 2, 260/230 ratio > 1.8.

#### Clariom™ S Assay-HT, Human Array Plate

Genome-wide expression profiling was done using the GeneChip^©^ WT PLUS assay kit and processed on the GeneTitan using Human Clariom S Assay-HT 16-Array Plate at the High-Throughput Genomics Wellcome Trust Centre for Human Genetics (Oxford, UK). RNA quality was controlled using Agilent Tape Station. The latest version of Affymetrix Genechip Command Console software for GeneTitan was used for quality check and data analyses.

### Statistical analysis

For Study 1, continuous variables were tested for normal distribution using the Kolmogorov–Smirnov test. Non-normally distributed variables are presented as median [25th–75th percentile], whereas normally distributed variables are presented as mean ± SD. The correlation of two continuous variables was evaluated with Pearson’s *r* or Spearman’s rank correlation coefficients, as appropriate. Continuous variable comparisons were performed using Student’s *t*-test or Mann–Whitney *U*-test (for two groups, as appropriate) or by ANOVA (for >2 groups), followed by Bonferroni correction for multiple comparisons. Power calculations were based on myocardial NADPH oxidase activity and we estimated that with 363 patients we could detect a difference of 0.25 in log(NADPH-stimulated $O_{2}^{.-}$) between high vs. low myocardial *SGLT1* expression with power 0.9 [assuming a standard deviation of 1.04 in log(NADPH-stimulated $O_{2}^{.-}$)].

To test whether the association of myocardial NADPH-stimulated $O_{2}^{.-}$ with myocardial *SGLT1* expression was independent of other risk factors, we performed multivariable linear regression analysis where myocardial NADPH-stimulated $O_{2}^{.-}$ was used as a dependent variable and myocardial *SGLT1* expression, age, sex, diabetes, hypertension, hypercholesterolaemia, and NYHA class were used as independent variables. The standardized betas (Bstand) are presented for each covariate.

In order to assess the independent effect of myocardial *SGLT1* expression on NADPH-stimulated and Vas2870-inhibitable $O_{2}^{.-}$ as well as myocardial *TNFα, IL6, ANP, BNP*, and *COL1A1* expression, each of the aforementioned variables was used as a dependent variable in individual multivariable linear regression analyses, and myocardial *SGLT1* expression was used as an independent variable. Age, sex, diabetes, hypertension, smoking (categorized as previous/active smoker vs. never-smoked) and NYHA class (class IV vs. lower classes) were used as covariates.

For Study 2, we estimated that with a minimum of five pairs of samples we would be able to identify a change of log(NADPH-stimulated $O_{2}^{.-}$) by 0.37 with α = 0.05, power 0.9 and SD for paired response difference of 0.30 upon canagliflozin treatments. Paired comparisons between two groups were performed using Wilcoxon signed-rank tests. When the changes between two paired analyses were compared that was performed using two-way ANOVA with interaction terms as stated in the respective figure legends. All tests were two-sided and alpha (α) was set at 0.05. Statistical analysis was performed using SPSS version 20.0 and R version 3.6.0.

## Results

### SGLT1/2 expression profile in the human heart

**Figure 1:**
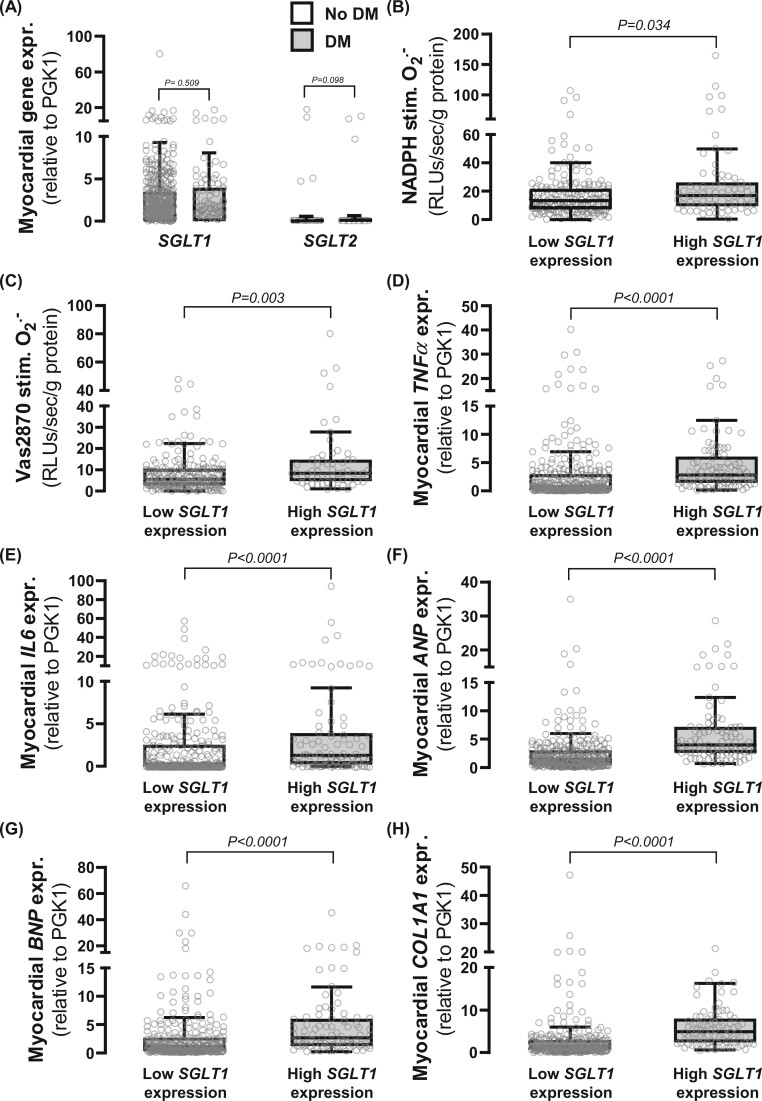
Sodium-glucose cotransporter (SGLT)1/2 expression in human atrial myocardium and relations with myocardial redox state and inflammation biomarkers. (*A*) *SGLT1* was abundantly expressed in the human atrial myocardium, contrary to *SGLT2*. *n* = 357 [290 non-diabetic patients (no DM); 67 diabetic patients (DM)]. *SGLT1* expression was positively correlated with NADPH-stimulated (*B*) and Vas2870-inhibitable (*C*) $O_{2}^{.-}$ as well as tumour necrosis factor-α (*TNFα, D*), interleukin-6 (*IL6, E*), atrial natriuretic peptide (*ANP, F*), brain natriuretic peptide (*BNP*, G), and collagen 1A1 (*Col1A1, H*) expression. *P*-values by Mann–Whitney *U*-test for no DM vs. DM (*A*) and highest quartile of *SGLT1* expression vs. rest (*B–H*). Data are presented as mean ± SD (*A*) and median [25th–75th percentile] (*B–H*).

We first evaluated the gene expression levels of *SGLT1* and *SGLT2* in the human myocardium (Study 1). *SGLT2* was undetectable in ∼96% of tested human myocardial samples whilst *SGLT1* was abundantly expressed (*Figure 1A* and Supplementary material online, *Figure S2*). *SGLT1* expression in atrial myocardium was positively associated with NADPH-stimulated (*Figure 1B*) and Vas2870-inhibitable $O_{2}^{.-}$ (*Figure 1C*) in 244 biopsies. Importantly, myocardial *SGLT1* expression was positively related to NADPH oxidase activity, independently of traditional cardiac risk factors (*Table 2*). In a multivariable linear regression analysis (log-transformed values), *SGLT1* expression in the human atrial myocardium was positively associated with basal $O_{2}^{.-}$ and NADPH oxidase activity independently of left ventricular ejection fraction and NYHA class (markers of heart-pumping function), suggesting an independent redox association with SGLT1 expression (Supplementary material online, *Table* *S1*). A multivariable linear regression analysis also revealed that the association between atrial SGLT1 expression and NADPH-stimulated $O_{2}^{.-}$ (stand.β: 0.449, *P* < 0.001) was independent of medical treatment with hypoglycaemic agents (stand.β: 0.202, *P* = 0.031), insulin (stand.β: 0.025, *P* = 0.791), statins (stand.β: −0.007, *P* = 0.940), angiotensin-converting enzyme inhibitor/angiotensin receptor blockers (stand.β: 0.081, *P* = 0.388), calcium channel blockers (stand.β: −0.117, *P* = 0.2), diuretics (0.092, *P* = 0.301), beta-blockers (stand.β: −0.048, *P* = 0.603), nitrates (stand.β: −0.026, *P* = 0.780), and antiplatelet therapy (stand.β: 0.046, *P* = 0.618).

**Table 2:** Table Multivariable regression model of myocardial NADPH oxidase activity

Covariate	Standardized β	Adjusted *P*-value
Myocardial SGLT1 expression	**0.339**	**0.001**
Age (years)	**0.152**	**0.021**
NYHA class	**0.137**	**0.032**
Smoking	0.067	0.284
Diabetes	0.054	0.378
Hypertension	0.022	0.737
Sex	−0.052	0.401
Hypercholesterolaemia	−0.034	0.601

SGLT1, sodium-glucose cotransporter 1. Statistically significant values are in bold.

S*GLT1* expression in atrial myocardium was also strongly related with markers of myocardial inflammation [tumour necrosis factor-α (*TNFα*), *Figure 1D* and interleukin-6 (*IL6*), *Figure 1E*], wall stretch [atrial natriuretic peptide (*ANP*), *Figure 1F* and brain natriuretic peptide (*BNP*), *Figure 1G*], as well as fibrosis [collagen 1A1 (*COL1A1*) expression, *Figure 1H*]. We also ran multivariable regression analyses to explore whether *SGLT1* gene expression was related to each one of these readouts independently of the baseline demographic characteristics. Indeed, *SGLT1* expression was related to myocardial *IL6* expression (stand.β: 0.110, *P* = 0.042), myocardial *TNFα* expression (stand.β: 0.133, *P* = 0.014), myocardial *ANP* expression (stand.β: 0.342, *P* < 0.001), myocardial *BNP* expression (stand.β: 0.161, *P* = 0.003), myocardial *COL1A1* expression (stand.β: 0.290, *P* < 0.001), NADPH-stimulated $O_{2}^{.-}$ (stand.β: 0.196, *P* = 0.003), and Vas2870-delta $O_{2}^{.-}$ (stand.β: 0.163, *P* = 0.014), independently of age, sex, smoking, diabetes, hypertension, and NYHA class.

### Direct effect of SGLT inhibitors on human myocardial redox state

**Figure 2:**
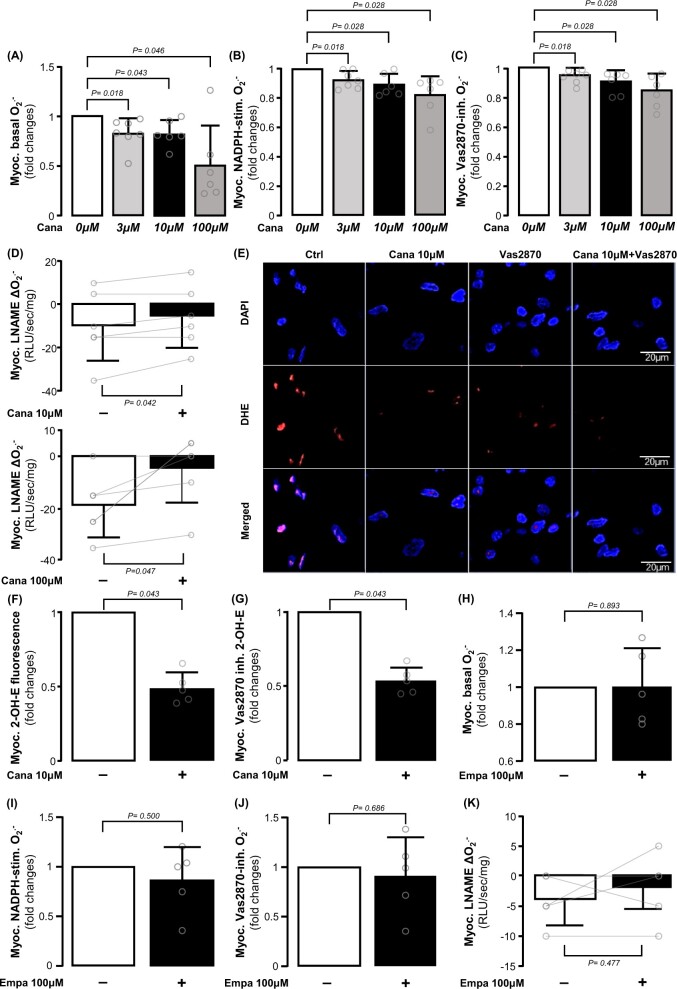
Direct effects of canagliflozin and empagliflozin on human myocardial redox state. *Ex vivo* canagliflozin (3, 10, and 100 μM) treatment for 1 h reduced basal (*A*), NADPH-stimulated (*B*), and Vas2870 inhibitable $O_{2}^{.-}$ (*C*) and increased L-NAME-(delta $O_{2}^{.-}$) (*D*) dose-dependently in human atrial myocardium. Canagliflozin decreased the intensity of basal and Vas2870 inhibitable 2-hydroxyethidium (2-OH-ethidium) fluorescence (*E–G*) in human atrial biopsies stained with dihydroethidium (DHE). Empagliflozin had non-significant direct effect on either of these measures (*H–K*). *n* = 5–7 in panels *A–L*. Data are presented as mean ± SD. *P*-values are calculated by Wilcoxon signed-rank test (*A–C, F–J*) and paired *t*-test (*D, K*).

Given the previous associations of myocardial *SGLT1* expression with oxidative stress, we explored the direct effects of canagliflozin, an SGLT2 inhibitor with significant affinity to SGLT1,^30^ on the redox state in the human heart. Incubation of human atrial myocardium with canagliflozin *ex vivo* reduced basal (*Figure 2A*), NADPH-stimulated (*Figure 2B*) and Vas2870-inhibitable (*Figure 2C*) $O_{2}^{.-}$ generation; importantly, all canagliflozin concentrations (3, 10, or 100 μM) had significant effects on these readings of myocardial redox state. In addition, canagliflozin reduced $O_{2}^{.-}$ production from uncoupled NOS, as estimated by the L-NAME-induced reduction in $O_{2}^{.-}$ (*Figure 2D*). Canagliflozin reduced oxidative stress in myocardial tissue from patients with or without diabetes mellitus, but it showed stronger effects on the myocardial tissue from diabetic patients (Supplementary material online, *Figure S3*). Canagliflozin reduced total and Vas2870-inhibitable dihydroethidium fluorescence in human atrial myocardium (*Figure 2E–G*), similar to the chemiluminescence experiments. In contrast, treatment with the SGLT2 inhibitor empagliflozin, which has little affinity to SGLT1,^31^ did not affect myocardial $O_{2}^{.-}$ generation, NADPH oxidase activity, or NOS coupling (*Figure 2H–K*). This finding could be explained by the very low affinity of empagliflozin to SGLT1, which is the main SGLT isoform in the human atrial and ventricular myocardium, and implies that clinically used SGLT2 inhibitors may exert direct myocardial effects depending on the degree of SGLT1 affinity. Therefore, we focused our next experiments on evaluating the direct effects of canagliflozin on myocardial redox signalling in humans, due to its dual SGLT1/SGLT2 affinity.

**Figure 3:**
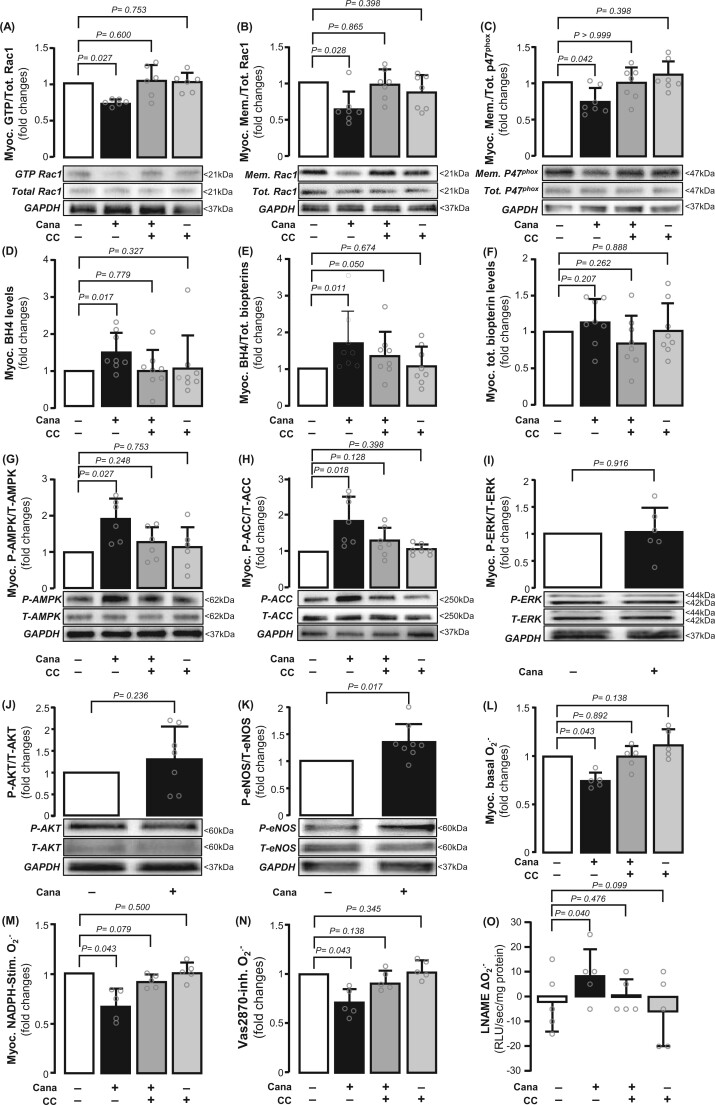
Effects of canagliflozin on myocardial NADPH oxidase activity and nitric oxide synthase (NOS) coupling status in the human atrial myocardium. Canagliflozin (10 μM for 1 h) inhibited GTP activation (*A*) and membrane translocation (*B*) of Rac1, as well as the membrane translocation of p47phox (*C*). Canagliflozin increased myocardial BH4 but not total biopterin content (*D–F*). Canagliflozin induced AMPK Thr172 phosphorylation (*G*) and downstream acetyl-coA carboxylase (ACC) Ser79 phosphorylation (*H*). These were prevented by the AMPK inhibitor, compound C (CC) (*G* and *H*). Canagliflozin did not affect ERK or AKT phosphorylation (*I* and *J*). Canagliflozin induced NOS Ser1177 phosphorylation (*K*). Compound C prevented the effects of canagliflozin on Rac1 activation, BH4 bioavailability (*A-F*), $O_{2}^{.-}$ generation (*L–N*), and NOS coupling (*O*). *n* = 5–8 pairs in panels *A–O*. Data are presented as mean ± SD. *P*-values are calculated by Wilcoxon signed-rank test (*A–N*) and paired *t*-test (*O*).

We first investigated the mechanism by which canagliflozin affects NADPH oxidase activity and NOS coupling status. To understand how canagliflozin reduced NADPH oxidase activity, we explored which subunits of the enzyme are mostly affected. Canagliflozin prevented the GTP activation of Rac1 (an important Nox1/2 subunit, *Figure 3A*) and its membrane translocation (*Figure 3B*), which lead to Nox1/2 isoform activation, while preventing the membrane translocation of the p47^phox^ subunit of Nox2 (*Figure 3C*). To assess whether NADPH oxidase activity increased $O_{2}^{.-}$ generation, not only directly, but also indirectly, we evaluated the oxidation of the NOS co-factor BH4. Canagliflozin increased BH4 levels in the atrial myocardium without affecting total biopterin content (*Figure 3D–F*), suggesting that it decreased BH4 oxidation (e.g. as a result of NADPH oxidase inhibition) rather than increased the biopterin biosynthetic pathway. Indeed, we also observed a reduction in BH2, the direct oxidation product of BH4, while biopterin, the terminal oxidation product of BH2 oxidation, was borderline decreased (Supplementary material online, *Figure S4A–F*). This would lead to improved enzymatic coupling of NOS and to a reduction in NOS-derived $O_{2}^{.-}$.

We next explored the upstream mechanism of the effects of canagliflozin on the myocardial redox state. Canagliflozin induced rapid phosphorylation of AMPKα2 (the main isoform in the heart) at the activation site Thr172 (*Figure 3G*), resulting in increased AMPKα2 activity documented by phosphorylation of its downstream target ACC at Ser79 (*Figure 3H*). Canagliflozin did not affect ERK phosphorylation at Thr202/Tyr204 or AKT at Ser473 (*Figure 3I and J*). Interestingly, canagliflozin also induced endothelial NOS phosphorylation at the activation site Ser1177 (*Figure 3K*), whereas it did not affect inducible NOS phosphorylation (Supplementary material online, *Figure S4G* and *H*). We next used CC, as an allosteric modulator of AMPK activity,^32^ to prove the concept that downstream SGLT1 effects are prevented by inhibiting AMPK activity. Compound C inhibited ACC phosphorylation (*Figure 3G and H*) as well as the ability of canagliflozin to inhibit Rac1 activation or increase BH4 bioavailability (*Figure 3A–E*). Compound C prevented the canagliflozin-induced reduction of myocardial $O_{2}^{.-}$ generation (*Figure 3L–N*) and of the increase in NOS coupling (*Figure 3O*), identifying AMPKα2 as a link between canagliflozin, NADPH oxidase activity, and NOS coupling. Canagliflozin could also reduce apoptosis and mitochondrial dysfunction through AMPKα2 (Supplementary material online, *Figure S5*).

### Direct effects of canagliflozin on primary human cardiomyocytes

**Figure 4:**
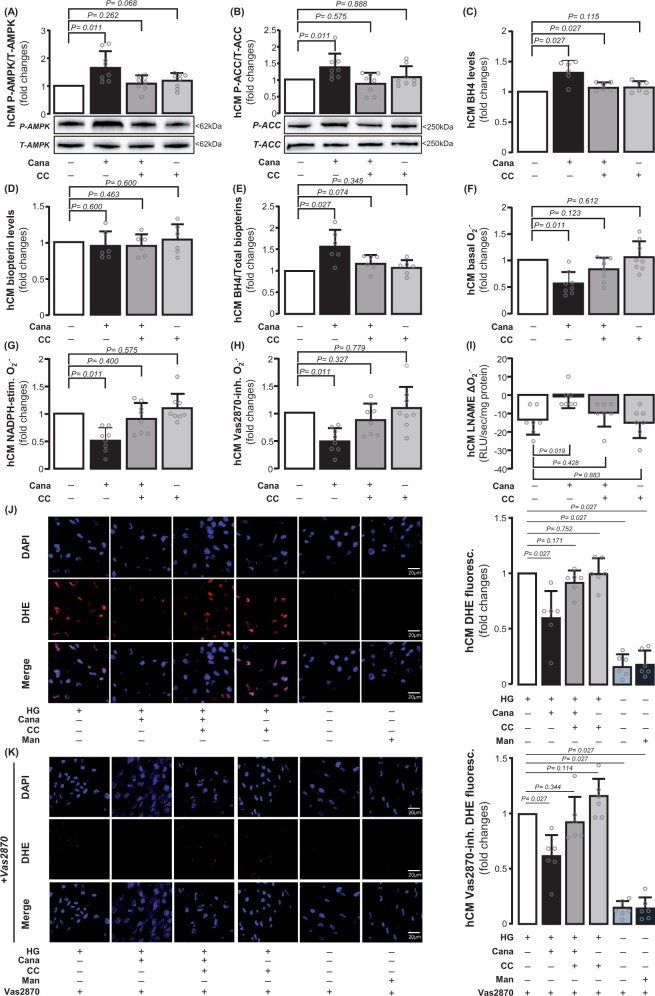
Direct effects of canagliflozin on human cardiomyocytes (hCM). Canagliflozin (10 μΜ) induced phosphorylation of AMPK and acetyl-coA carboxylase (ACC) in high glucose (HG)-treated human cardiomyocytes (*A* and *B, n* = 8) and increased BH4 levels without affecting total biopterin levels (*C–E, n* = 8). Canagliflozin decreased basal, NADPH-stimulated and Vas2870-inhibitable $O_{2}^{.-}$ and increased the value of L-NAME delta($O_{2}^{.-}$) in human cardiomyocytes (*F–I, n* = 7). Dihydroethidium (DHE) staining combined with Vas2870 confirmed these (*J–L, n* = 8). Data are presented as mean ± SD. *P*-values are calculated by Wilcoxon signed-rank test (*A–H, J, K*) and paired *t*-test (*I*).

We next studied the effects of canagliflozin specifically on primary terminally differentiated hCM (Supplementary material online, *Figure S1*). To mimic the *ex vivo* human atrial myocardium experimental conditions as well as a clinically relevant diabetic environment, hCM were cultured in high-glucose medium. Osmolality changes associated with high glucose had no effect on myocardial redox state using mannitol as osmolality control (Supplementary material online, *Figure S6*). As observed in the human atrial myocardium, canagliflozin increased AMPKα2 Thr172 phosphorylation (*Figure 4A*) leading to AMPKα2 activation, as assessed by the phosphorylation status of ACC at Ser79 (*Figure 4B*). In addition, canagliflozin increased BH4 bioavailability, without altering total biopterins in hCM, in a CC-reversible way (*Figure 4A–E*). Similarly to the human atrial myocardium, exogenous canagliflozin reduced basal $O_{2}^{.-}$ generation (*Figure 4F*) and NADPH oxidase activity (*Figure 4G and H*) and improved NOS coupling (*Figure 4I*) in an AMPKα2-dependent manner (since all those effects were reversed by CC) in hCM. Dihydroethidium staining confirmed the effect of canagliflozin on NADPH oxidase-derived O_2_.^−^ in hCM (*Figure 4J–L*). Canagliflozin also induced NOS phosphorylation at its activation site Ser1177 in hCM (Supplementary material online, *Figure S7*). These findings were replicated in H9c2 cells (Supplementary material online, *Figures S8* and *S9*), where the results were more striking after culture in a high-glucose medium.

### The role of SGLT1 in myocardial redox regulation by canagliflozin

**Figure 5:**
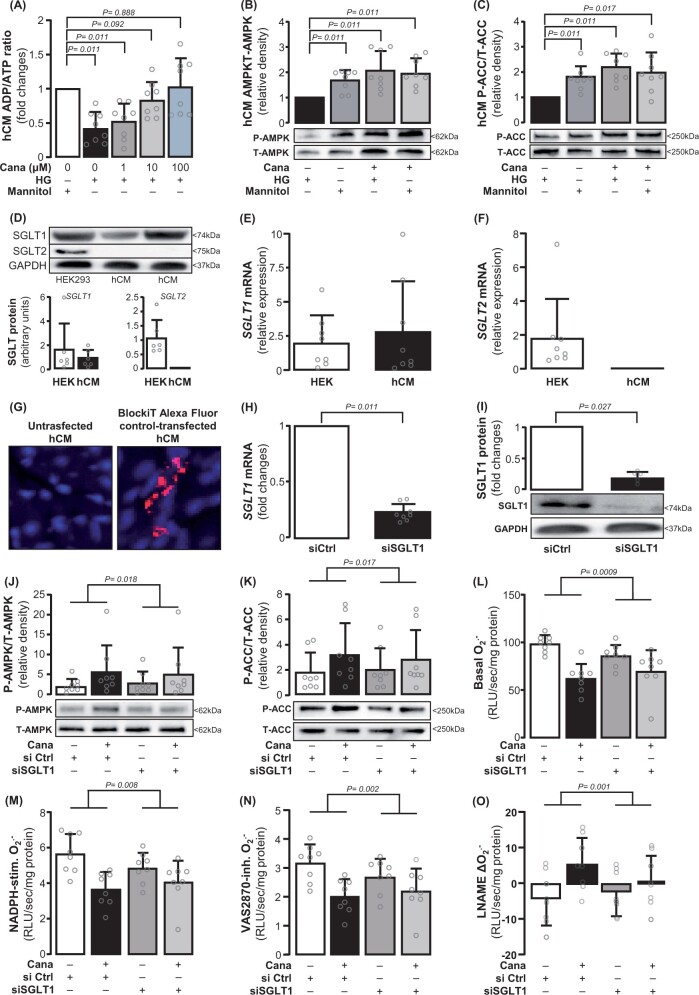
SGLT1 mediates the effects of canagliflozin on myocardial redox state. Canagliflozin increased ADP/ATP dose-dependently in human cardiomyocytes, while glucose deprivation from the culture medium (NG) induced similar changes in ADP/ATP ratio (*A, n* = 8). There were no differences in AMPK and ACC phosphorylation among the NG-incubated cells with or without canagliflozin (10μΜ), and high glucose (HG)-incubated cells with canagliflozin (panels *B* and *C, n* = 8). *SGLT1* expression was detected, while *SGLT2* was not detected in human cardiomyocytes (*D–F, n* = 8). *SGLT1* was knocked down using siRNA (transfection low toxicity and efficiency was confirmed by transfecting BlockiT Alexa Fluor Red Fluorescent control, *G*), resulting into ∼76% down-regulation of *SGLT1* mRNA (panel *H, n* = 6), and ∼85% protein down-regulation (panel *I, n* = 6). Canagliflozin-induced AMPK and ACC phosphorylation was attenuated in *SGLT1* knocked down human cardiomyocytes compared with siRNA ctrl human cardiomyocytes (*J* and *K, n* = 8). SGLT1 deletion diminished canagliflozin-induced decrease of basal (*L*), NADPH-stimulated (*M*), and Vas2870-inhibitable $O_{2}^{.-}$ production (*N*) and increased L-NAME delta $O_{2}^{.-}$ (*O*). *n* = 8 in *L–O*. Data are presented as mean ± SD. *P*-values are calculated by Wilcoxon signed-rank test (*A–C, H, I*). Comparisons in canagliflozin responses between siControl and siSGLT1 cells were performed with two-way ANOVA with treatment (canagliflozin) × cell type (siControl or siSGLT1) interaction (*J–O*).

To explore how canagliflozin affects the upstream phosphorylation of AMPKα2, we next evaluated the intracellular ADP-to-ATP ratio in hCM, which can allosterically regulate AMPKα2 activity and auto-phosphorylation. Canagliflozin increased ADP/ATP in a dose-dependent manner (*Figure 5A*). Depleting glucose from the culture media resulted in increased ADP/ATP (*Figure 5A*) and activated AMPKα2, evidenced by ACC phosphorylation. Adding canagliflozin to glucose-starved hCM did not lead to further change on either AMPKα2 or ACC (*Figure 5B and C*). These indicate that the effect of canagliflozin on hCM is dependent on glucose uptake, which then affects intracellular ADP/ATP and subsequent AMPKα2 activation.

Consistent with our findings in human cardiac samples, hCM *SGLT2* expression was undetectable (*Figure 5D–F*). *SGLT1* expression was confirmed by qPCR and Western immunoblotting using HEK293 cells as positive control (*Figure 5D and E*). To further explore the dependency of the effects of canagliflozin on SGLT1, we knocked down the expression of *SGLT1* in hCM using siRNA (*Figure 5G*), achieving down-regulation of *SGLT1* mRNA by ∼76% and protein by ∼85% (*Figure 5H and I*). This prevented canagliflozin-induced AMPK activation and downstream ACC phosphorylation in hCM (*Figure 5J and K*). *SGLT1* knock down prevented the ability of canagliflozin to reduce basal, NADPH-stimulated and Vas2870-inhibitable $O_{2}^{.-}$ (*Figure 5L–N*), supporting that canagliflozin suppresses myocardial NADPH oxidase activity by inhibiting SGLT1. *SGLT1* knock down in hCM also prevented the ability of canagliflozin to improve NOS coupling (*Figure 5O*). *SGLT1* knock down did not lead to a compensatory increase in *SGLT2* expression (Supplementary material online, *Figure S10*). Interestingly, knock down of SGLT1 did not fully mimic the effects observed with canagliflozin, and this could be explained either by the incomplete SGLT1 knock down technically achieved in these cells, or even by the presence of off-target effects of canagliflozin, independent of SGLT1/2 inhibition.

### Direct effects of canagliflozin on redox-sensitive pro-inflammatory signalling in the human myocardium

**Figure 6:**
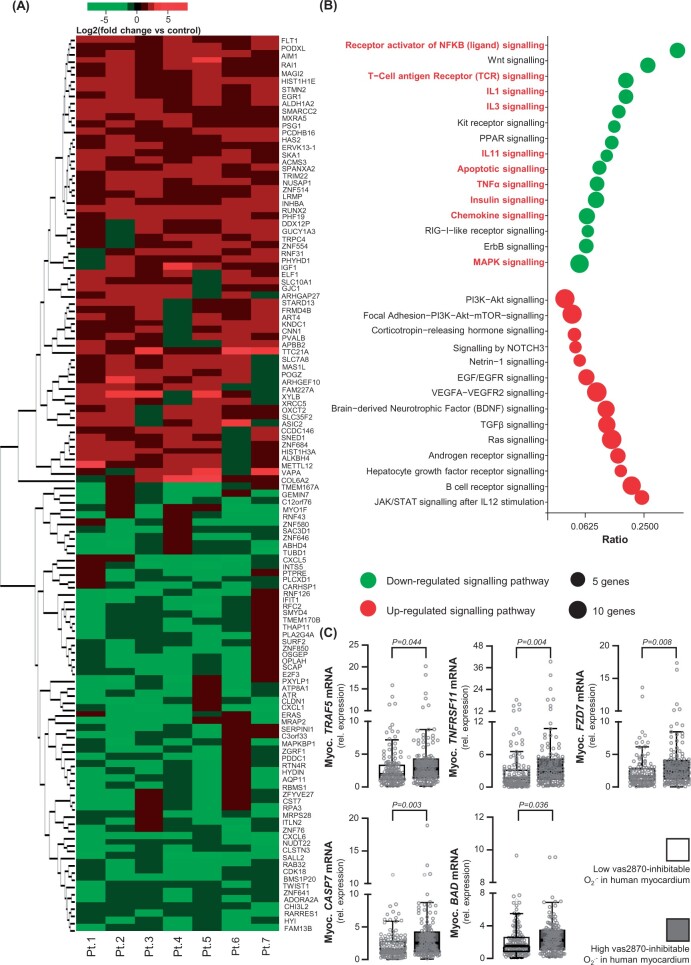
Canagliflozin has a global anti-inflammatory and anti-apoptotic effect on human cardiomyocytes. In this experiment, human cardiomyocytes were cultured in high-glucose medium (25 mM) for 72 h and then treated with canagliflozin (10 μM) or DMSO for 24 h. Heat map of 127 down- or up-regulated genes by canagliflozin (fold change >1.5 or `−1.5, *P* < 0.05) in canagliflozin-treated human cardiomyocytes from *n* = 7 patients (*A*). NFkB, Wnt, IL1, IL3, TNFα, chemokine, MAPK pathways, and apoptotic pathways (highlighted by red font) were down-regulated by canagliflozin (more than 50% of pathway genes down-regulated by canagliflozin, *B*). *TNFRSF11, TRAF5, FZD7, CASP7*, and *BAD* were the most down-regulated individual genes upon canagliflozin treatment, implicated in NFkB, TNFα, and apoptosis pathways. The mRNA expression of these genes was positively correlated with myocardial NADPH oxidase activity (i.e. high Vas2870-inhibitable signal) in 240 atrial biopsies from Study 1 (*C*). Data are presented as median [25th–75th percentile]. *P*-values are calculated by Mann–Whitney *U*-test for high (above median) vs. low (below median).

After establishing a role for canagliflozin as a regulator of the myocardial redox state, we explored its downstream effects on the transcriptomic profile of hCM. Canagliflozin (10 μM) treatment for 24 h altered the expression of 946 differentially expressed genes (DEGs, 466 up-regulated and 480 down-regulated) in hCM, with 127 DEGs demonstrating an effect size of >50% or `−50% (*Figure 6A*). Pathway analysis revealed that canagliflozin down-regulated a number of pro-inflammatory pathways such as those of IL1, IL3, TNFα, chemokines, MAPK signalling, and others, as well as apoptotic signalling pathways (*Figure 6B*). Notably, the nuclear factor kappa beta (NFkB) signalling pathway, a major redox-sensitive inflammatory transcriptional regulator, was one of the most down-regulated targets by canagliflozin, suggesting that it may contribute to the antioxidant effect of canagliflozin in hCM. Cell survival pathways such as *PI3K-Akt, NOTCH3*, and *Ras* signalling were also up-regulated by canagliflozin. Key genes of the *NFkB, TNF-α*, and apoptotic pathways mostly affected by canagliflozin included *TNFRSF11, TRAF5, FZD7, CASP7*, and *BAD*. The expression of these genes, quantified in atrial biopsies of 364 patients from Study 1, was positively associated with myocardial NADPH oxidase activity (*Figure 6C*).

## Discussion

In this work, we identify SGLT1 as the main SGLT isoform in the human myocardium, its expression being positively related with myocardial oxidative stress. As the main objective of this study was to evaluate the direct effects of SGLT1 vs. SGLT2 inhibition on the myocardium and SGLT2 is not expressed in human myocardium and cardiac myocytes, we focused on canagliflozin, which has significant affinity for SGLT1. We further demonstrate that a clinically relevant concentration of canagliflozin directly suppresses NADPH oxidase activity, increases BH4 bioavailability, and improves NOS coupling in isolated hCM and myocardial tissue via an SGLT1/AMPK/Rac1-dependent mechanism. These suppress redox-sensitive pro-inflammatory and pro-apoptotic signalling in the human cardiomyocyte, providing a novel mechanism contributing to the beneficial effects of canagliflozin on cardiac adverse events (*Graphical abstract*).

The role of SGLT isoforms in myocardial physiology is controversial.^33^^,^^34^  *SGLT2* expression has been detected in hCM after exposure to high glucose; however, other studies have shown no *SGLT2* expression in human hearts. In contrast, SGLT1 was found in hCM ^34^^,^^35^ while functionally damaging variants in *SGLT1* have been associated with reduced death/heart failure risk.^36^ We definitively demonstrate, using a large number of human myocardial biopsies and hCM, that SGLT1 is abundantly expressed in the human heart, whereas SGLT2 is barely detectable.

Clinically used SGLT2 inhibitors have variable SGLT1 affinities. Canagliflozin displays higher SGLT1 affinity than other SGLT2 inhibitors,^30^^,^^31^^,^^37^ whilst empagliflozin is the most selective SGLT2 inhibitor.^31^ Evidence has suggested differing cellular responses to canagliflozin vs. empagliflozin, e.g. canagliflozin is a stronger inducer of AMPK phosphorylation in HEK293 cells or human vascular endothelial cells.^13^^,^^38^ Importantly, we demonstrate for the first time that canagliflozin (in concentrations comparable to *in vivo* pharmacological levels^39^^,^^40^) directly inhibits NADPH oxidase-derived oxidative stress in human myocardial redox signalling via SGLT1/AMPK/Rac1 signalling, which may be more potent than empagliflozin. Furthermore, canagliflozin could reverse the decrease in ADP/ATP ratio due to a hyperglycaemic environment by inhibiting glucose uptake in hCM.

Myocardial oxidative stress facilitates apoptosis and inflammation, leading to cardiac fibrosis and promoting cardiac dysfunction.^41^ Myocardial inflammation is influenced by redox-sensitive transcriptional factors such as NFkB.^42^ Our transcriptome analysis demonstrated that canagliflozin suppresses several redox-sensitive pro-inflammatory and pro-apoptotic pathways in hCM, such as those of TNF-α and IL-1 as well as, crucially, on NFkB activity. The link of canagliflozin with myocardial redox regulation was further validated in ∼400 human atrial biopsies, by demonstrating a clear relationship between myocardial NADPH oxidase-derived $O_{2}^{.-}$ and the expression of key genes regulated by canagliflozin.

Certain aspects of the interplay between SGLT2 inhibitors and myocardial disease are not explored in our work. SGLT2 inhibitors with little SGLT1 selectivity (e.g. empagliflozin) also demonstrate cardioprotective effects despite lacking SGLT1 affinity. A very recent study from Kolijn *et al*.^14^ described an antioxidant effect of empagliflozin, in humans with heart failure with preserved ejection fraction as well as in ZDF obese rats, evaluated by lipid peroxidation levels, which was accompanied by improved NO-sGC-PKG signalling. Although in our study empagliflozin did not affect NADPH oxidase-derived superoxide production in human atrial myocardium, these two pieces of work may describe distinct mechanisms, which could be complementary and the discrepancies in results may be due to different participant characteristics, use of different (indirect) readings of redox state (we specifically targeted NADPH oxidase-derived superoxide, whilst Kolijn *et al.* ^14^ targeted lipid peroxidation, which is influenced by reactive oxygen species levels, antioxidant capacity, and cell metabolism) and tissue type differences (atrial vs. ventricular tissue). Furthermore, this suggests that systemic SGLT2 inhibition has also indirect effects on the human heart, most likely by affecting kidney function as well as the secretome of other tissues with indirect, endocrine effects on cardiac function, which is not explored here. However, some SGLT2 inhibitors (like canagliflozin) may well have chronic direct effects on the heart via cardiac SGLT1 inhibition, and this direct effect on the human myocardium was the objective of our study. This concept warrants further validation in appropriate *in vivo* animal models, such as *Sglt1* loss- and gain-of-function mouse models.

Of note is the fact that the *ex vivo* experiments were performed on myocardial tissue of atrial origin, whereas our mechanistic experiments were carried out in cardiomyocytes derived from ventricles. The fact that our proposed mechanism is shown in a similar way in all models (human atrial myocardium, human ventricular cardiomyocytes, and terminally differentiated H9c2 cells) further strengthens the validity of the proposed mechanisms by which canagliflozin affects myocardial biology. In this study, we evaluate the membrane translocation of NADPH oxidase subunits by measuring their levels on isolated membranes vs. the cytosolic phase using an ultra-centrifugation protocol, rather than electron microscopy, and this is a methodological limitation that needs to be acknowledged. Finally, in translational studies like this one, surgical human tissue availability varies, and this has led to variability in sample sizes across the various experiments in this study.

In conclusion, we demonstrate for the first time that canagliflozin inhibits NADPH oxidase activity and improves NOS coupling via SGLT1/AMPKα2/Rac1 signalling, whilst suppressing several pro-inflammatory and pro-apoptotic pathways in the human myocardium. Our work describes an important SGLT1-mediated mechanism that could contribute to the cardioprotective effects of the SGLT2 drug class.

## Supplementary material

Supplementary material is available at *European Heart Journal* online.

## Supplementary Material

ehab420_Supplementary_Data

## Data Availability

The data underlying this article will be shared on reasonable request to the corresponding author.
