# Supplementary material for: Effects of canagliflozin on human myocardial redox signalling: clinical implications
Source: Eur Heart J. 2021 Jul 19;42(48):4947–60. doi: 10.1093/eurheartj/ehab420 (PMC8691807; doi:10.1093/eurheartj/ehab420)
Supplement: ehab420_Supplementary_Data [file ehab420_supplementary_data.docx]

**-Supplemental material-**

**Effects of Canagliflozin on Human Myocardial Redox Signalling: Clinical Implications**

Hidekazu Kondo^1,2^, Ioannis Akoumianakis^1^, Ileana Badi^1^, Nadia Akawi^1^, Christos P Kotanidis^1^, Murray Polkinghorne^1^, Ilaria Stadiotti^3^, Elena Sommariva^3^, Alexios S Antonopoulos^1^, Maria C Carena^1^, Evangelos K Oikonomou^1^, Elsa Mauricio Reus^1^, Rana Sayeed^4^, George Krasopoulos^4^, Vivek Srivastava^4^, Shakil Farid^4^, Surawee Chuaiphichai^1^, Cheerag Shirodaria^5^, Keith M Channon^1,4^, Barbara Casadei^1^, Charalambos Antoniades^1,4,6*^

^1^Division of Cardiovascular Medicine, Radcliffe Department of Medicine, University of Oxford, Oxford, UK

^2^Department of Cardiology and Clinical Examination, Faculty of Medicine, Oita University, Oita, Japan

^3^Unit of Vascular Biology and Regenerative Medicine, Centro Cardiologico Monzino IRCCS, Milan, Italy

^4^Oxford University Hospitals NHS Trust, Oxford, UK

^5^Caristo Diagnostics, Oxford UK

^6^Acute Vascular Imaging Centre, University of Oxford, Oxford, UK

**Short title:** Effects of Canagliflozin on Human Heart

***Corresponding author**:

Charalambos Antoniades MD PhD FRCP FESC

Professor of Cardiovascular Medicine University of Oxford

Division of Cardiovascular Medicine, L6 West Wing, John Radcliffe Hospital,

Headley Way, Oxford OX3 9DU, UK

e-mail: [antoniad@well.ox.ac.uk](mailto:antoniad@well.ox.ac.uk)

Tel: +441865228340

Fax: +44186534615

**Supplemental methods**

**Human Tissue Harvesting**

Human atrial myocardial segments were collected from the cannulation site during surgery in ice-cold, Krebs HEPES Buffer (NaCl 99 mM, KCl 4.7 mM, MgSO_4_ 1.2 mM, KH_2_PO4 1 mM, CaCl_2_ 1.9 mM, NaHCO_3_ 25 mM, Glucose 25 mM, NaHEPES 20 mM, PH 7.35) and immediately transferred to the lab on ice. Attached epicardial fat was carefully removed, and atrial myocardial tissue was washed in ice-cold Krebs HEPES buffer and cut into thin strips containing all myocardial layers, as previously described ^1^. The strips were either snap-frozen and kept at -80^o^C for baseline phenotyping or used for *ex vivo* experiments.

***Ex Vivo* Incubations of Human Atrial Myocardium**

To examine the direct effects of Canagliflozin on myocardial O_2_^.-^ production, human myocardial tissue from the right atrium appendage was incubated *ex vivo* for 1 hour with/without Canagliflozin in the presence/absence of the pharmacological inhibitor of AMPK, compound C (CC), or with carrier. Briefly, human atrial myocardial specimens were cut into strips as described above, and strips were first equilibrated for 20 minutes in Krebs HEPES buffer pH 7.35 at 37°C and then incubated for 1 hour in the presence or absence of Canagliflozin 10 μmol/L and 100 μmol/L (25mg; Cayman, MI, USA) ± CC (10 μmol/L) or carrier (DMSO). Incubated samples were snap-frozen in -80^o^C and used at a later stage for myocardial O_2_^.-^ measurements and downstream signalling experiments, as described later.

The majority of the *ex vivo* and *in vitro* experiments were performed using a canagliflozin concentration of 10μM (some were done with 100μM as stated in appropriate sections). This concentration has been used in previous mechanistic studies^2^ and it is comparable to the *in vivo* peak concentration of canagliflozin achieved with common clinical doses ^3^. Furthermore, this concentration is also comparable to its IC50 for SGLT1, corresponding to a physiologically relevant effect via SGLT1 inhibition ^4^.

**Superoxide quantification**

Myocardial O_2_^.-^ production was measured in the human atrial myocardium using lucigenin (5μmol/L)-enhanced chemiluminescence, as we have previously described ^1, 5^. Myocardial tissue was homogenised in ice-cold Krebs HEPES Buffer (pH 7.35) in the presence of protease inhibitor (Roche Applied Science, IN, USA) using a pre-cooled Polytron homogeniser. Protein concentration was quantified and unified to 0.2 mg before the experiment.

The contribution of NADPH-oxidase activity to myocardial O_2_^.-^ production was quantified in the presence of NADPH at 100μmol/L. Vas2870 (400 μmol/L; Sigma Aldrich, a specific pan-NADPH-oxidase inhibitor) was used to obtain the Vas2870-inhibitable O_2_^.-^ signal, which constitutes a more specific index of NADPH-oxidase activity as previously reported ^1^.

The contribution of uncoupled NOS to myocardial O_2_^.−^ was evaluated by using the NOS inhibitor L-NAME (at 1 mM). Baseline readings were first acquired, then 20 min L-NAME incubations were performed in the homogenates and the baseline reading was subtracted from the reading at the end of the L-NAME incubation (delta-LNAME O_2_^.−^) as we have previously described ^6^.

O_2_^.-^ was also quantified in cell lysates. Following *in vitro* incubations, cells were scraped in ice-cold Krebs HEPES Buffer (pH=7.35) in the presence of protease inhibitor (Roche Applied Science) by scraping. The lysates were sonicated and processed similarly to human atrial myocardial homogenates as described above in order to quantify the various O_2_^.-^ sources.

**RNA Isolation and Quantitative Real Time-Polymerase Chain Reaction (qRT-PCR)**

***RNA isolation***

Total RNA was isolated by a phenol to chloroform (1:5 ratio) separation protocol followed by a magnetic beads-based RNA purification method on a KingFischer magnetic particle processor (Thermo Fischer Scientific), using the MagMAX mirVana total RNA isolation kit (Thermo Fischer Scientific, Catalogue Number A27828). RNA concentration and integrity were assessed spectrophotometrically on NanoDrop ND-1000 as recently described ^7^.

***Reverse transcription***

RNA was reverse-transcribed to cDNA by using the SuperScript VILO mastermix (Thermo Fischer Scientific) following the manufacturer’s instructions and extending the cDNA synthesis step to two hours at 60^o^C on a Veriti thermal cycler (ABI).

***Quantitative real-time PCR***

Quantitative real-time PCR was performed by TaqMan chemistry, using the standard universal TaqMan protocol as indicated by the Manufacturer, on a QuantStudio 7 flex real-time PCR system (Thermo Fischer Scientific). All samples were run in duplicates using 5 ng of cDNA as starting mass, and data was analysed by the Pfaffl method ^8^. PGK1 was used as housekeeping gene for human atrial myocardial tissue and hCMs. The IDs of the TaqMan probes used are: *PGK1*: Hs00943178_g1; *SGLT1*: Hs01573790_m1; *SGLT2*: Hs00894634_g1, Hs00894635_g1, Hs00894639_g1, Hs00894641_g1, Hs00894644_g1, Hs00894646_g1; *TRAF5*: Hs01072219_m1; *TNFRSF11*: Hs00921372_m1; *FZD7*: Hs00942807_s1; *CASP7*: Hs00169152_m1, *BAD*: Hs00188930_m1

***Cohort-wide gene expression analyses***

Target gene and housekeeping gene Ct values were quantified by qRT-PCR. Furthermore, the same standards were run on every qPCR plate to extrapolate the amplification efficiency per plate. Target gene and housekeeping gene Ct values were quantified and used to quantify target copy numbers with endogenous housekeeping correction (Efficiency^–ΔCt^). This corrected value was then normalised to the highest standard of the standard curve, providing a corrected expression value relative to the highest standard (Efficiency^–ΔΔCt^). Hence the provided values are housekeeping-normalised expressions relative to a single reference sample (the highest standard of the standard curve), allowing for accurate interpolate analysis.

**Sample Preparation for Western Blotting**

Human atrial myocardial tissue was homogenised in ice-cold RIPA buffer (Cell Signalling) supplemented with proteases and phosphatases inhibitors (Cell Signalling) by using a pre-cooled Polytron homogeniser. Protein concentrations were determined using the BCA assay (Pierce), with albumin as standard.

For the cell culture experiments, protein extracts were obtained from 100% confluent differentiated cells, preceded by washes in cold PBS and lysis in RIPA buffer (Cell Signalling) containing protease and phosphatase inhibitors (Cell signaling). ULTRARIPA Kit (Funakoshi Japan) was used to detect SGLT1 and SGLT2, as this kit provides more efficient extraction of membrane proteins such as SLGT1 and SGLT2. Protein concentrations were determined using the BCA assay (Pierce), using albumin as the standard.

**Western Blotting**

20μg of denatured protein extracts were analysed by SDS/PAGE on 4-12% polyacrylamide gradient gel, and the proteins were then transferred to nitrocellulose membranes (Amersham). For detection of immuno-reactive bands, ECL select Western Blotting Detection Reagent (Amersham) was used. Western blots were quantified using Image Lab Bio-rad software integrated density analysis. The primary antibodies used were: anti-SGLT1 (#MBS540266, MyBioSource, 1:250), anti-SGLT2 (#MBS544866, MyBioSource, 1:250), p-AMPKα2, Thr172 (#2532, Cell Signalling, 1:1000), AMPKα2 (#5832, Cell Signalling, 1:1000), phospho(Ser79)-acetyl-CoA carboxylase (ACC) (#3661, Cell Signalling, 1:1000), ACC (#3676, Cell Signalling, 1:1000), p-AKT, Ser473 (#4060, Cell Signalling, 1:1000), AKT (#4691, Cell Signalling, 1:1000), p-NOS, Ser1177 (#612393, BD bioscience, 1:1000), NOS (#610296, BD bioscience, 1:1000), p-ERK1/2, Thr202/Tyr204 (#4370, Cell Signalling, 1:1000), ERK1/2 (#4695, Cell Signalling, 1:1000), mouse anti-GAPDH HRP-conjugated (#G9295, Sigma 1:20,000). HRP-conjugated anti-rabbit (#A9169, Sigma, 1:10,000) and HRP-conjugated anti-mouse (#A9044, Sigma, 1:15,000) were used as secondary antibodies as appropriate.

**Rac1 activation assay**

Rac1 activation was detected using an active rac1 detection kit (Cell Signalling). Human atrial myocardial tissue samples were homogenised, using the lysis buffer provided by the kit, supplemented with protease and phosphatase inhibitors. Lysates (500ug) were then incubated with glutathione resin and GST-PAK1-PBD according to the manufacturer’s specific instructions (Cell Signalling), and GTP-proteins were precipitated and isolated. Western blot analysis was performed using mouse monoclonal anti-Rac1 (#05-389, Millipore, 1:1,000) primary antibody and HRP-conjugated anti-mouse (1:15,000) secondary antibody.

**Evaluation of myocardial Rac1 and p47phox membrane translocation**

Membrane translocation of Rac1 and p47phox in the human atrial myocardial tissue was estimated by differential centrifugation of myocardial homogenates to isolate membrane proteins,^9^ and membrane-translocated Rac1 or p47phox protein was determined by Western immunoblotting using mouse monoclonal anti-Rac1 (#05-389, Millipore, 1:1,000) and anti-p47phox (#4301, Cell Signalling, 1:1000) primary antibody and the corresponding secondary antibodies. Briefly, myocardial segments were homogenized in ice-cold HEPES buffer (HEPES 20 mM, 150 mM NaCl, and 1 mM EDTA, pH=7.4) supplemented with a protease inhibitor cocktail (Roche, UK). Debris was removed by centrifugation of the homogenates at 2,800 g at 4°C for 20 min, and the protein content of supernatants was evaluated by the Pierce BCA protein assay kit. 500 mg of total protein were adjusted to 200 μL for all samples, added into ultracentrifugation tubes and ultra-centrifuged at 100,000 g for 60 min at 4°C to separate the cytosolic from the membrane proteins. Following removal of supernatants containing the cytosolic proteins, pellets were re-suspended in 35 μL of lysis buffer containing 1% Triton and left for 20 min on ice. Protein concentrations were then quantified, and samples processed for Western immunoblotting as described previously ^1^.

**Measurement of Myocardial and Cardiomyocyte Biopterins**

Myocardial tetrahydrobiopterin (BH4), dihydrobiopterin (BH2), and biopterin levels were each determined separately from the same sample, using high-performance liquid chromatography followed by serial electrochemical and fluorescent detection, as we have previously described^6^. Total biopterin levels are the results of the sum of BH4, BH2, and biopterin individual levels. Results were expressed per total protein for each sample.

**Oxidative Fluorescent Microtopography**

*In situ* O_2_^.-^ was determined in human atrial myocardial cryosections and hCMs with oxidative fluorescent dihydroethidium (DHE, D23107, Thermo-Fisher) as previously described ^1^.

Myocardial samples were subsequently snap frozen in OCT compound. Cryosections (30 μm) were incubated with DHE (5 μmol/L for 30 minutes) in Kreps-Hepes buffer, with or without Vas2870 (400 μmol/L). Fluorescence images were obtained by using confocal microscope (63x, Zeiss LSM 510 META laser scanning confocal microscope). DHE fluorescence was quantified by using ImageJ software, while all analyses were performed in a blinded fashion.

Incubated hCM were directly incubated with DHE (5 μmol/L for 30 minutes), and washed by PBS three times. Fluoroshield Mounting Medium with DAPI (ab104139, Abcam) was used for counterstaining. Fluorescence image acquisition and analysis were performed similarly to the myocardial tissue as described above.

**TUNEL assay**

Human atrial myocardial samples were fixed in 4% paraformaldehyde, embedded in paraffin, cut into 5 µm sections, and stained. Apoptotic cells were detected with the *in situ* terminal deoxynucleotidyl transferase-mediated dUTP nick end-labelling (TUNEL) method using an apoptosis kit (Medical Biological Lab, Nagoya, Japan), as described previously^10^. Briefly, sections were treated with proteinase K and then treated with a mixture of terminal deoxynucleotidyl transferase (TdT), fluorescein isothiocyanate-dUTP, and TdT buffer II at 37°C for 1 h. After washing the slides with PBS, they were mounted with mounting medium containing 4′6-diamidino-2-phenylindole (DAPI). The number of TUNEL-positive cell was calculated as a percentage of the total number of DAPI-labelled cells. Four images per atrium were analysed from 6 patients per group to obtain the mean values. Images were acquired and digitized on a BZ-9000 Biolevo epifluorescence microscope with an attached digital camera.

**JC-10 Mitochondrial Membrane Potential Assay**

Mitochondrial function of cells isolated from human right atrial appendages was analysed by means of flow cytometry, in Oita University (Japan), as previously described^11^. The cells were stained with the JC-10 Mitochondrial Membrane Potential Assay Kit (Flow Cytometry) (ab112133, Abcam), following the manufacturer’s instructions. After staining with 7-amino-actinomycin D (Sigma) to discriminate dead cells, flow cytometry was conducted on a FACSCalibur (BD Bioscience), and the data were analysed with Win MDI software.

**Measurement of intracellular ADP and ATP**

ATP/ADP ratio was quantified in hCM by a commercial ATP/ADP ratio kit (MAK135, Sigma) according to the manufacturer's protocol. Briefly, after removing the culture medium, 90 μL of ATP reagent was added to each well of a 96 well-plate where hCM were cultured. The plate was incubated for 1 minute at room temperature, then luminescence (relative light units) was read on a luminometer for the ATP assay. The plate was then incubated for 10 minutes and read for luminescence to provide the background prior to ADP measurement. Immediately following the reading, 5 mL of ADP Reagent was added to each well, and after 1 minute, luminescence was read. The ATP/ADP ratio was calculated as the corrected ratio of the ATP and ADP luminescence readings according to the manufacturer's protocol.

**Transfection studies with SGLT1 siRNA**

SGLT1 siRNA and negative control siRNA (Thermo Fisher Scientific) were used to knock-down SGLT1 in hCM and differentiated H9c2. Briefly, 2x10^5^ differentiated hCMs and confluent were incubated in high glucose medium in a 24-well plate for 72 hours, and medium was replaced by Optimem on the day of transfection. Lipofectamine RNAiMax (Thermo Fisher Scientific) was used to transfect hCM with 25 pmoles of siRNA according to the instructions provided by the manufacturer. 24 hours post-transfection, Optimem was replaced by high glucose-medium. To test the transfection ability as well as the toxicity of lipofectamine RNAiMax on hCM, BlockiT Alexa Fluor Red Fluorescent control (14750100, ThermoFisher) was used as positive transfection control. Differentiated H9c2 cells were incubated in high glucose medium in a 12-well plate for 72 hours, and medium was replaced by Optimem on the day of transfection. Lipofectamine RNAiMax (Thermo Fisher Scientific) was used to transfect 10 pmoles of siRNA or siRNA negative control according to the manufacturer’s instructions. 5 hours post-transfection, Optimem was replaced by high glucose-medium. The cells were cultured for additional 48 hours and processed for RNA extraction with RNeasy Mini Kit (Qiagen) and reverse transcription and quantitative real-time PCR were performed as described above.

**Supplementary Table 1:** Regression model of RAA NADPH-stimulated O_2_^.-^

| **Covariate** | **Bst** | **P-value** |
| --- | --- | --- |
| RAA SGLT1 expression | 0.150 | 0.021* |
| NYHA class | 0.145 | 0.026* |

RAA: Right atrium appendage; SGLT1: Sodium-glucose cotransporter 1; Bst: Standardised beta for each covariate

**Supplementary figures**

**
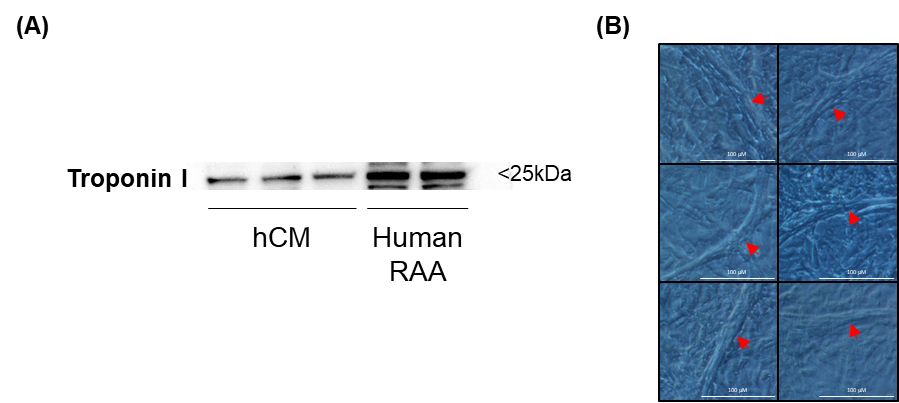
**

**Figure S1: Characterization for phenotype of hCM.** Troponin I expression in the fully differentiated human cardiomyocytes, was confirmed by Western blotting (A) by using human right atrial appendage (RAA) as a positive control. Myotube (red arrowheads) formation was confirmed morphologically (B).

**
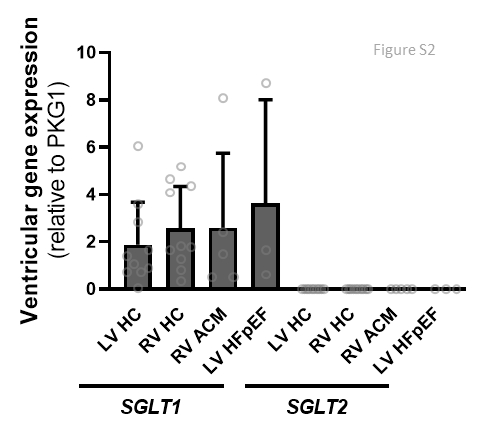
**

**Figure S2. SGLT1 is expressed in the human ventricular myocardium, contrary to SGLT2.** SGLT1 and SGLT2 mRNA levels were analysed by quantitative reverse transcriptase polymerase chain reaction and normalized to corresponding PGK1 levels in left and right ventricles from 10 healthy controls (LV HC and RV HC), in 5 right ventricles from patients with arrhythmogenic cardiomyopathy (RV ACM) and 3 interventricular septum (left side) from patients with heart failure with preserved ejection fraction (LV HFpEF). Data are presented as mean ± SD.

**
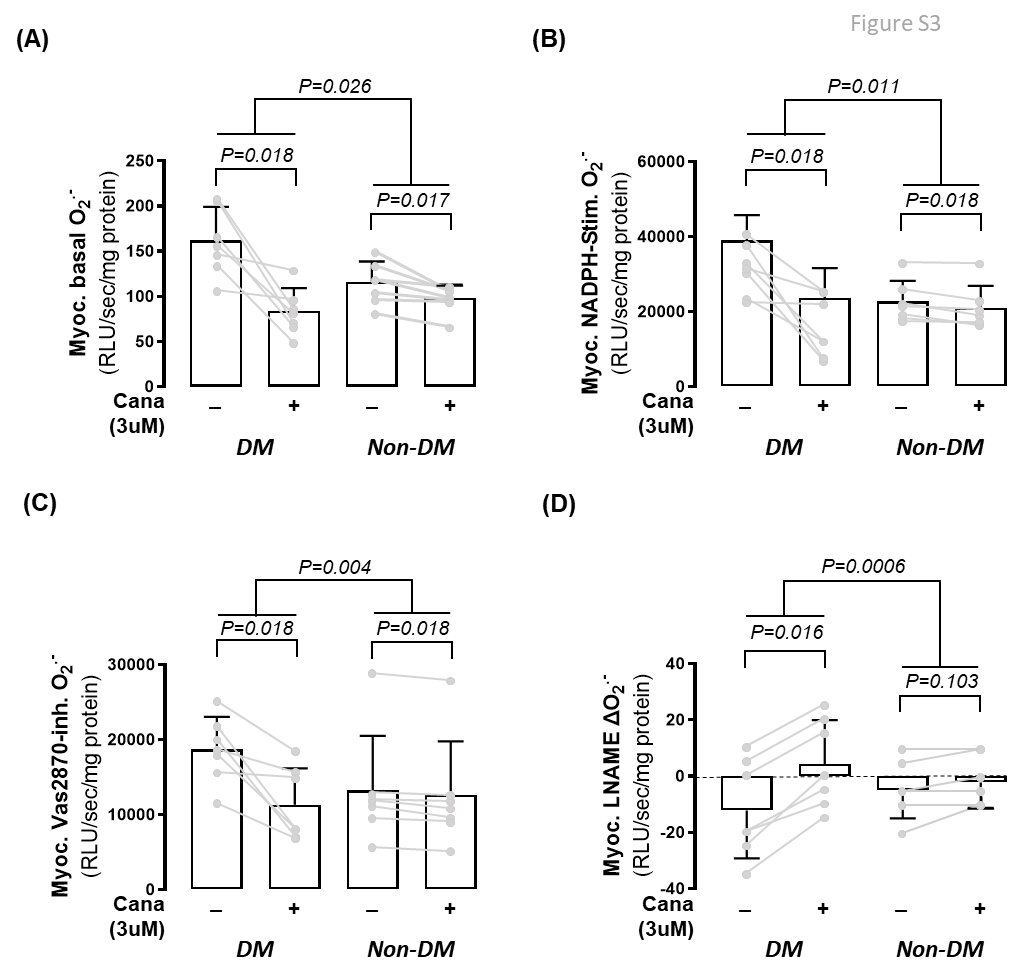
**

**Figure S3: Direct effects of canagliflozin on human myocardial redox state in diabetics vs. non-diabetics.** In human atrial myocardium, *ex vivo* canagliflozin (3 μM) treatment for 1h reduced basal (A), NADPH-stimulated (B), and Vas2870 inhibitable O2.- (C) to a greater extent in diabetics as compared to non-diabetics. Canagliflozin also increased L-NAME-(delta O_2_^.-^) (D) in diabetics but had no significant effect in non-diabetics. n=7 in panel A-D. Data are presented as mean ± SD. P-values are calculated by Wilcoxon signed-rank test for comparison between Cana- and Cana+ and Mann Whitney U test for comparisons between deltas.

**
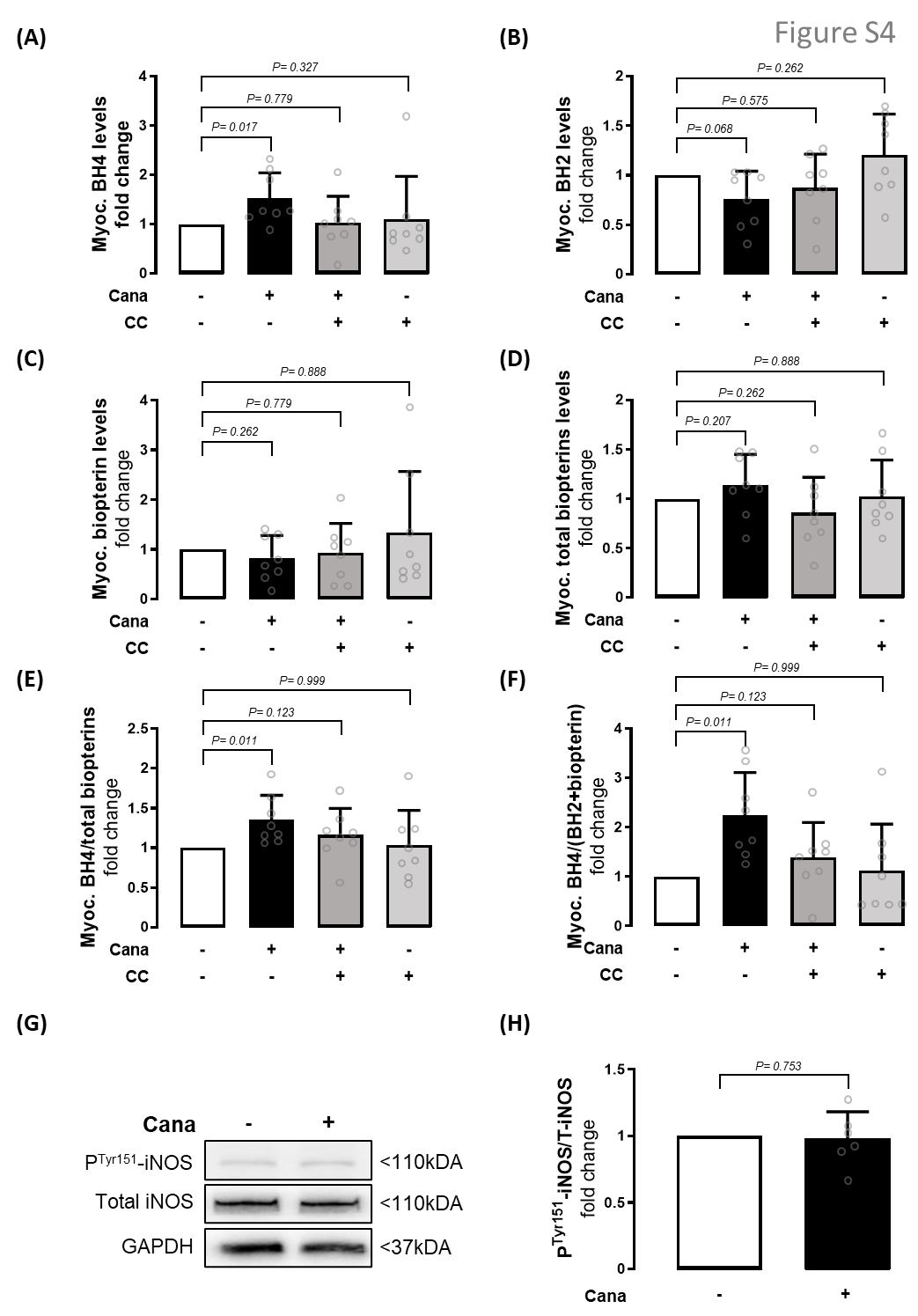
**

**Figure S4: Effects of canagliflozin on biopterin species contents and on phosphorylation of inducible nitric oxide synthase (iNOS) in the human myocardium.** Canagliflozin increased tetrahydrobiopterin (BH4) content (A) and decreased dihydrobiopterin (BH2) content (B) in the human right atrial appendage, without changes in biopterin or total biopterin species content (C-D). Effects of canagliflozin on BH4/total biopterins (E) and BH4/(BH2+biopterin) (F). The effects of canagliflozin on BH4 and BH2 were reversed by compound C (CC). (G-H) Canagliflozin (10μM for 1h) treatment of right atrial appendages did not change iNOS phosphorylation as analysed by Western Blot (n=6). Data are presented as mean ± SD. P-values are calculated by Wilcoxon signed-rank test.

**
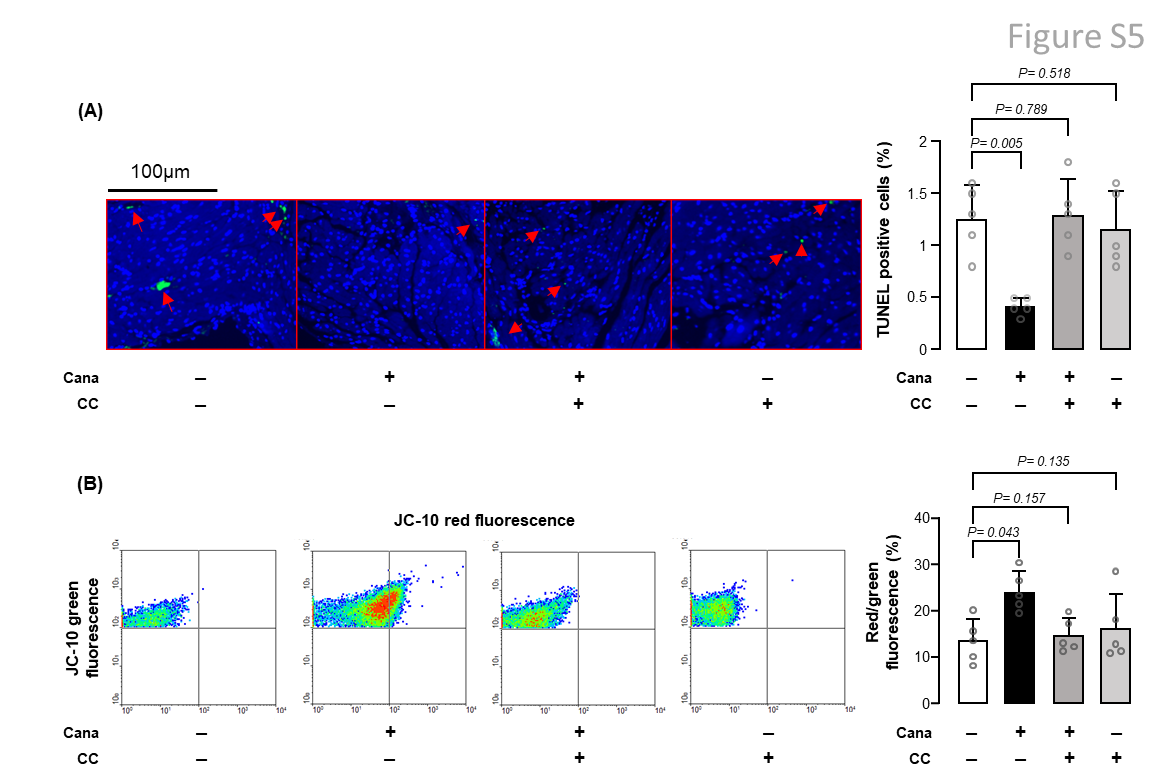
**

**Figure S5: Canagliflozin reduces apoptosis and mitochondrial dysfunction through AMPKα2.** Canagliflozin (10μM for 1h) reduced apoptosis (A) and mitochondrial dysfunction (B). These were prevented by the AMPK inhibitor, compound C (CC) (A, B). n=5. Data are presented as mean ± SD. P-values are calculated by paired t-test.

**
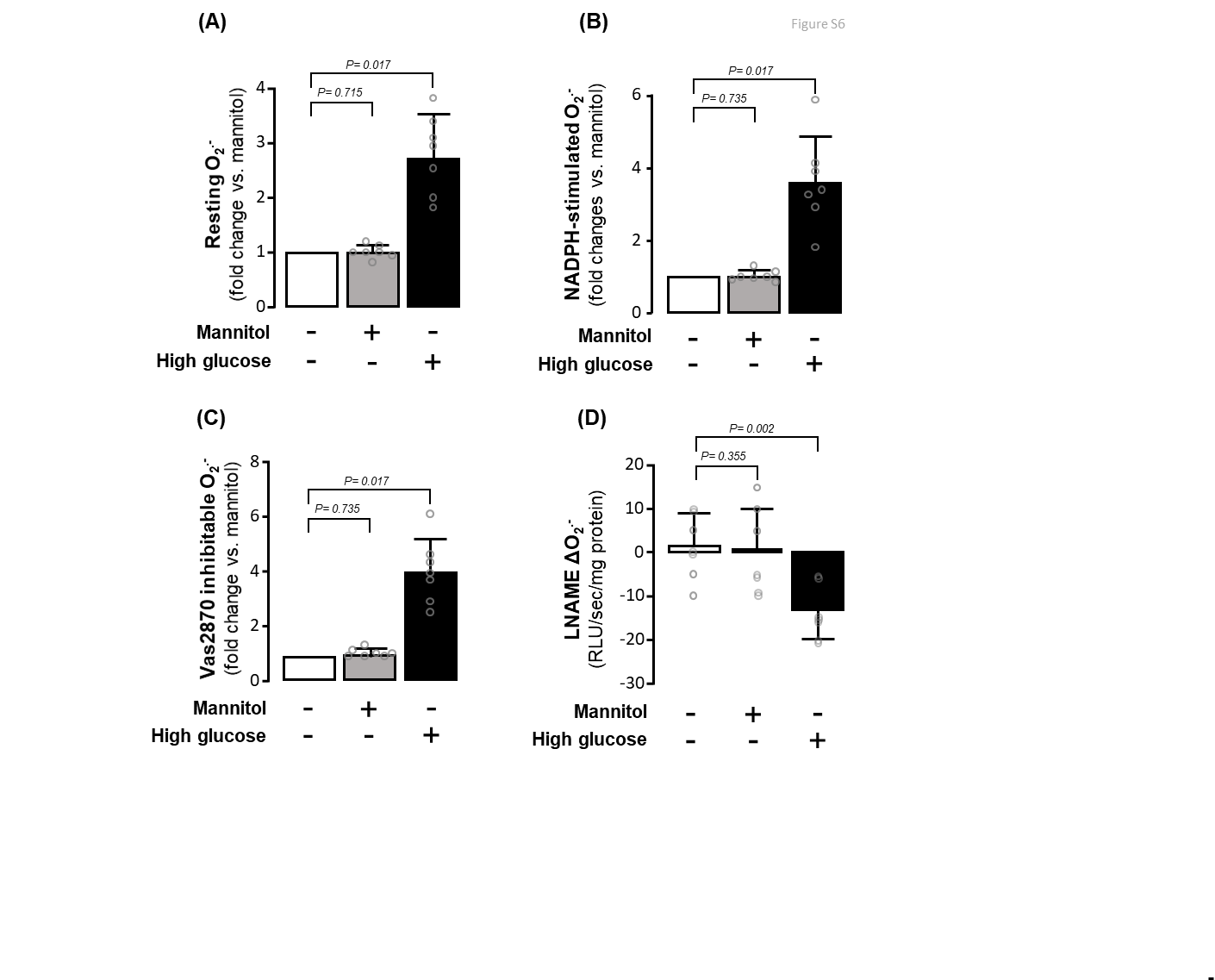
**

**Figure S6: The effect of osmolality changes associated with high glucose** Mannitol, used for osmolality control, did not have any effect on myocardial redox state, contrary to high glucose (A-D). Data are presented as mean ± SD. P-values are calculated by Wilcoxon signed-rank test (A-C) and paired t-test (D).

**
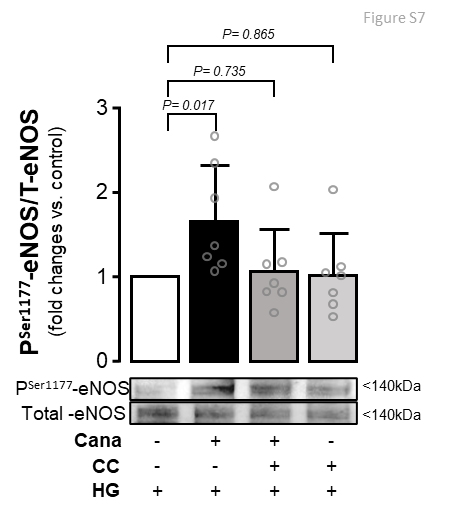
**

**Figure S7: Phosphorylation of eNOS in hCM in high glucose (HG) medium.** Canagliflozin increased the phosphorylation of e-NOS, which was diminished by compound C (CC) administration. n=6 of paired sample. Data are presented as mean ± SD. P-values are calculated by Wilcoxon signed-rank test.

**
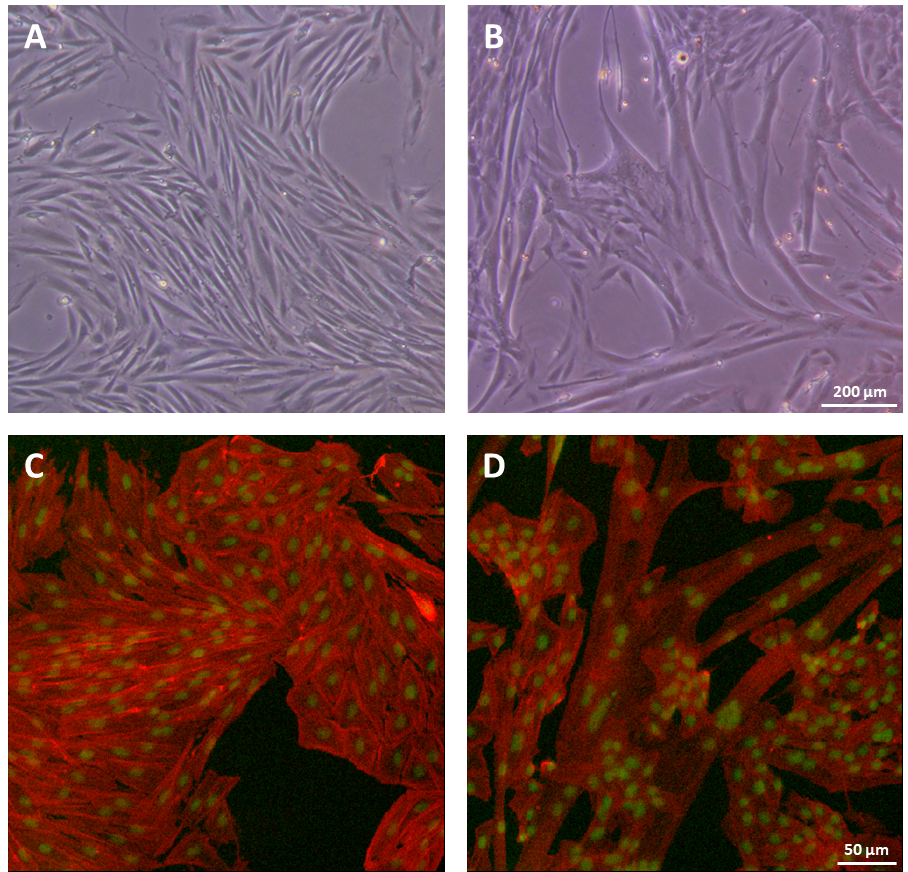
**

**Figure S8: Characterization of the differentiated H9c2 cardiomyocytes *in vitro* model.** Bright-field representative images of undifferentiated rat H9c2 cardiomyoblasts (A) and differentiated cardiomyocytes (B) obtained after growing confluent cardiomyoblasts for 7 days in differentiation medium. Representative images of undifferentiated cardiomyoblasts (C) and multinucleated myotubes at day 7 of differentiation (D) stained with the fluorescent dyes DAPI (for nuclei, in green) and Phalloidin-CF568 (for F-actin, in red).

**
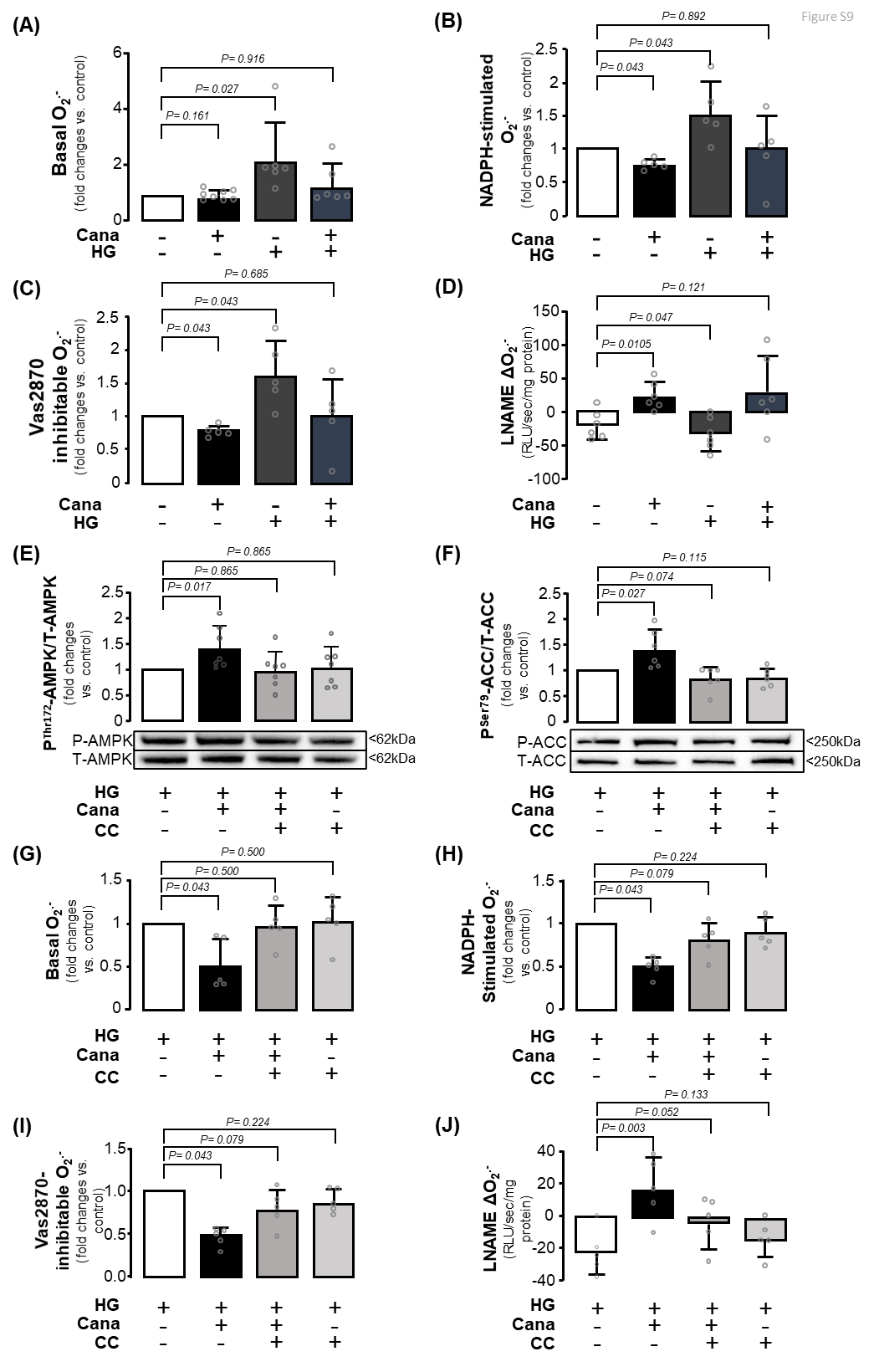
**

**Figure S9: Canagliflozin improved redox state in H9C2 cells, which was enhanced following incubation with high glucose, and this effect is dependent on AMPK signalling.** Canagliflozin reduced the basal (A), NADPH-stimulated (B), and Vas2870 inhibitable O_2_^.^**^-^** (C), and increased L-NAME delta O_2_^.-^ values (D). The effects were exaggerated in the cells treated with high glucose medium. (A-D). n=8 per experiment; data are presented as mean ± SD. Canagliflozin treatment induced phosphorylation of AMPK at the activation site Thr172 (E) and the downstream target acetyl-coA carboxylase (ACC), via phosphorylation at Ser79 (F). The observed effects on ACC phosphorylation, a marker of AMPK activity, were prevented by the AMPK inhibitor, compound C (CC) (E and F). AMPK inhibition by CC ameliorated the ability of canagliflozin to suppress the myocardial O_2_^.-^ generation (G-I) and improve the NOS coupling (L). n=6-8 of paired sample in panel E-L. Data are presented as mean ± SD. P-values are calculated by Wilcoxon signed-rank test (A-C, E-I) and paired t-test (D, J).


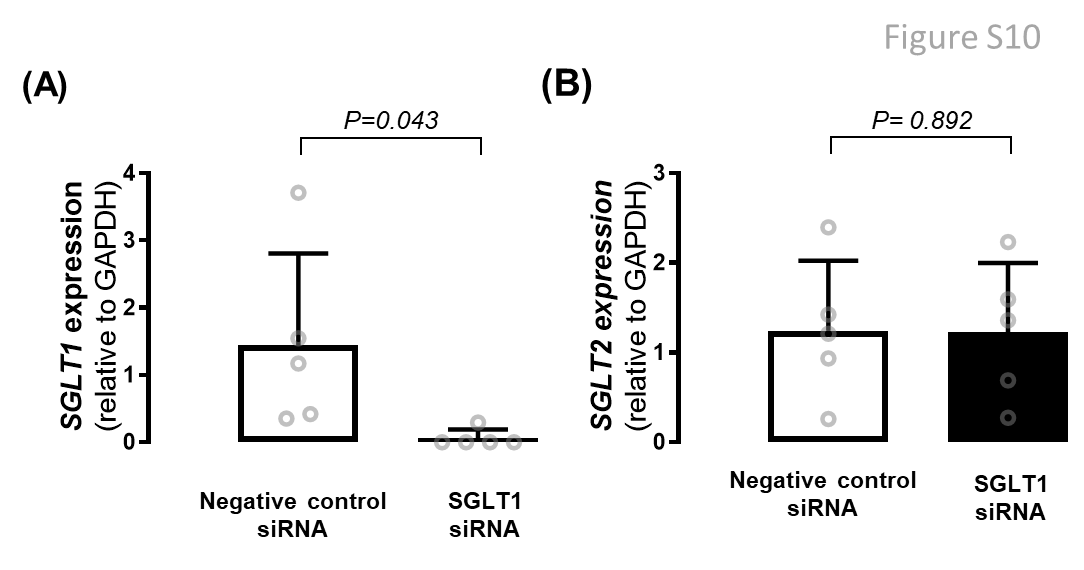


**Figure S10: SGLT1 knock down does not have any effect on SGLT2 expression in cardiomyocytes.** SGLT1 was knock down in H9c2 cells (A) and SGLT2 gene expression was evaluated (B). Data are presented as mean ± SD. P-values are calculated by Wilcoxon signed-rank test.

**Supplemental references**

1. Antonopoulos AS, Margaritis M, Verheule S, Recalde A, Sanna F, Herdman L, Psarros C, Nasrallah H, Coutinho P, Akoumianakis I, Brewer AC, Sayeed R, Krasopoulos G, Petrou M, Tarun A, Tousoulis D, Shah AM, Casadei B, Channon KM, Antoniades C. Mutual Regulation of Epicardial Adipose Tissue and Myocardial Redox State by PPAR-γ/Adiponectin Signalling. Circ Res 2016;**118**(5):842-55.

2. Hawley SA, Ford RJ, Smith BK, Gowans GJ, Mancini SJ, Pitt RD, Day EA, Salt IP, Steinberg GR, Hardie DG. The Na+/Glucose Cotransporter Inhibitor Canagliflozin Activates AMPK by Inhibiting Mitochondrial Function and Increasing Cellular AMP Levels. Diabetes 2016;**65**(9):2784-94.

3. Devineni D, Curtin CR, Polidori D, Gutierrez MJ, Murphy J, Rusch S, Rothenberg PL. Pharmacokinetics and pharmacodynamics of canagliflozin, a sodium glucose co-transporter 2 inhibitor, in subjects with type 2 diabetes mellitus. J Clin Pharmacol 2013;**53**(6):601-10.

4. Mudaliar S, Polidori D, Zambrowicz B, Henry RR. Sodium-Glucose Cotransporter Inhibitors: Effects on Renal and Intestinal Glucose Transport: From Bench to Bedside. Diabetes Care 2015;**38**(12):2344-53.

5. Reilly SN, Jayaram R, Nahar K, Antoniades C, Verheule S, Channon KM, Alp NJ, Schotten U, Casadei B. Atrial sources of reactive oxygen species vary with the duration and substrate of atrial fibrillation: implications for the antiarrhythmic effect of statins. Circulation 2011;**124**(10):1107-17.

6. Margaritis M, Antonopoulos AS, Digby J, Lee R, Reilly S, Coutinho P, Shirodaria C, Sayeed R, Petrou M, De Silva R, Jalilzadeh S, Demosthenous M, Bakogiannis C, Tousoulis D, Stefanadis C, Choudhury RP, Casadei B, Channon KM, Antoniades C. Interactions between vascular wall and perivascular adipose tissue reveal novel roles for adiponectin in the regulation of endothelial nitric oxide synthase function in human vessels. Circulation 2013;**127**(22):2209-21.

7. Akoumianakis I, Sanna F, Margaritis M, Badi I, Akawi N, Herdman L, Coutinho P, Fagan H, Antonopoulos AS, Oikonomou EK, Thomas S, Chiu AP, Chuaiphichai S, Kotanidis CP, Christodoulides C, Petrou M, Krasopoulos G, Sayeed R, Lv L, Hale A, Naeimi Kararoudi M, McNeill E, Douglas G, George S, Tousoulis D, Channon KM, Antoniades C. Adipose tissue-derived WNT5A regulates vascular redox signaling in obesity via USP17/RAC1-mediated activation of NADPH oxidases. Sci Transl Med 2019;**11**(510).

8. Pfaffl MW. A new mathematical model for relative quantification in real-time RT-PCR. Nucleic Acids Res 2001;**29**(9):e45.

9. Antonopoulos AS, Margaritis M, Coutinho P, Shirodaria C, Psarros C, Herdman L, Sanna F, De Silva R, Petrou M, Sayeed R, Krasopoulos G, Lee R, Digby J, Reilly S, Bakogiannis C, Tousoulis D, Kessler B, Casadei B, Channon KM, Antoniades C. Adiponectin as a link between type 2 diabetes and vascular NADPH oxidase activity in the human arterial wall: the regulatory role of perivascular adipose tissue. Diabetes 2015;**64**(6):2207-19.

10. Kohno H, Takahashi N, Shinohara T, Ooie T, Yufu K, Nakagawa M, Yonemochi H, Hara M, Saikawa T, Yoshimatsu H. Receptor-mediated suppression of cardiac heat-shock protein 72 expression by testosterone in male rat heart. Endocrinology 2007;**148**(7):3148-3155.

11. Kondo H, Abe I, Gotoh K, Fukui A, Takanari H, Ishii Y, Ikebe Y, Kira S, Oniki T, Saito S. Interleukin 10 treatment ameliorates high-fat diet–induced inflammatory atrial remodeling and fibrillation. Circulation: Arrhythmia and Electrophysiology 2018;**11**(5):e006040.
